# Paternal Circadian Disruption Impairs Offspring Cognition via Sperm microRNAs

**DOI:** 10.1002/advs.202514510

**Published:** 2026-04-28

**Authors:** Kexin Zou, Sisi Luo, Binliang Tang, Zhiqiang Liu, Yuchuan Zhou, Yicong Meng, Zhengmu Wu, Zheng Sun, Weihui Shi, Jianzhong Sheng, Chuanjin Yu, Zhenhua Li, Siyi Wei, Mo Zhang, Yujie Luo, Jing Yan, Xueyun Qin, Jiahang Mo, Chengliang Zhou, Jinsong Li, Hefeng Huang, Yifu Ding, Guolian Ding

**Affiliations:** ^1^ Institute of Reproduction and Development Shanghai Key Laboratory of Reproduction and Development Obstetrics and Gynecology Hospital Fudan University Shanghai China; ^2^ Shanghai First Maternity and Infant Hospital Tongji University Shanghai China; ^3^ Center for Rehabilitation Medicine Rehabilitation & Sports Medicine Research Institute of Zhejiang Province Department of Rehabilitation Medicine Zhejiang Provincial People's Hospital Affiliated People's Hospital Hangzhou Medical College Hangzhou Zhejiang China; ^4^ Shanghai Institute of Biochemistry and Cell Biology Center for Excellence in Molecular Cell Science Chinese Academy of Sciences University of Chinese Academy of Sciences Shanghai China; ^5^ The International Peace Maternity and Child Health Hospital Shanghai Jiao Tong University School of Medicine Shanghai China; ^6^ Department of Medicine – Endocrinology Baylor College of Medicine Houston Texas USA; ^7^ Institute of Medical Genetics and Development Key Laboratory of Reproductive Genetics (Ministry of Education) and Women's Hospital Zhejiang University School of Medicine Zhejiang China; ^8^ Shanghai Key Laboratory of Female Reproductive Endocrine Related Diseases Shanghai China; ^9^ Key Laboratory of Systems Health Science of Zhejiang Province School of Life Science Hangzhou Institute for Advanced Study University of Chinese Academy of Sciences Hangzhou China

**Keywords:** cognition, epigenetics, non‐coding RNAs, offspring health, paternal circadian disruption

## Abstract

A father's lifestyle can impact offspring's health. However, it is unclear whether and how paternal circadian rhythm disruption affects offspring neural development and cognitive function. Here, we show that male mice exposed to constant light (LL‐F0) led to memory dysfunction and impaired hippocampal synaptic plasticity in male, but not female, F1 offspring (LL‐F1). Paternal circadian disruption altered expression profiles of small RNAs, especially microRNAs (miRNAs), in mouse sperm. These miRNA changes were associated with chromatin accessible regions in spermatogonial stem cells, pointing to an early germline origin of the disrupted epigenetic state. In human sperm, we identified and validated upregulation of miR‐92a‐3p. Microinjection of total small RNAs from LL‐F0 sperm or synthetic miR‐92a‐3p/miR‐25‐3p into zygotes was sufficient to phenocopy offspring cognitive impairment, whereas inhibition of these miRNAs in zygotes partially reversed the phenotype. These findings support a role for sperm‐borne miRNAs in the intergenerational transmission of neurobehavioral deficits induced by paternal circadian disruption.

## Graphical abstract

Paternal circadian disruption remodels the sperm small RNA payload, elevating miR‐92a‐3p/miR‐25‐3p levels and perturbing early embryonic gene regulatory programs. Microinjection experiments and single‐embryo transcriptomics reveal sex‐specific developmental vulnerabilities, ultimately impairing offspring hippocampal synaptic plasticity and cognition. These findings uncover a germline mediated mechanism linking disturbed paternal rhythms to intergenerational neurodevelopmental outcomes.

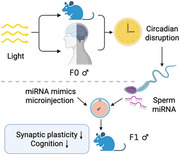

## 1 Introduction

The mammalian circadian rhythm system, which maintains about 24 h rhythmicity of all molecular and physiological process, is an important coordinator of motivated behaviors [1, 2]. Disruption of normal circadian rhythm may lead to metabolic, reproductive, and neurological disorders [3, 4, 5, 6, 7]. At the whole‐organism behavioral level, the behavioral rhythm is controlled by the central nervous system and is directly entrained by light that is perceived by the eye [8, 9]. Light pollution has become a potential health risk factor, nighttime illumination either from indoor light pollution or the use of electronic devices such as mobile phones before bedtime, has been associated a greater risk for depressive symptoms [10, 11]. Exposure to dim light‐at‐night (LAN) induced depressive‐like behaviors in hamsters and mice [12, 13, 14, 15], which effect may be mediated by a neural pathway from retinal melanopsin‐expressing ganglion cells to the dorsal perihabenular nucleus (dpHb) to the nucleus accumbens (NAc) [16]. Even acute exposure to low‐level LAN is sufficient to induce neurological changes and depressive‐like behavior [17]. These illustrate the challenges our increasingly globalized and 24‐h society faces in balancing mental health with convenience and the increased productivity of modern lifestyle.

Except for the impact on the individual's health, increasing attention has been raised in the impact of parental exposure to the adverse environmental effect on offspring. Abnormal circadian rhythm can affect gene‐environment interactions [18]. Previous studies have shown that the health of the offspring may be affected by maternal circadian disruption during pregnancy [19, 20, 21, 22, 23]. The effects observed under the maternal paradigm may be due to altered intrauterine developmental environment, maternal endocrine or behaviors. By comparison, after exposing to adverse environment, breeding the exposed males with control females in normal environment involves fewer confounding factors, providing an efficient way to explore the intergenerational effects mediated by germ cells. Recently, some studies have paid attention to the paternal effects such as nutrition or stress on offspring [24, 25, 26, 27, 28, 29, 30]. It is noteworthy that except very few studies reported that depressive‐like behavior in offspring [31], whether paternal circadian rhythm disturbances affect the cognition in offspring is lack of research.

Epigenetic signatures such as DNA methylation, histone modifications, and noncoding RNAs can be transmitted to the next generation through the germ line [29, 32, 33]. Although nucleosomes are widely replaced by protamine in mature sperm, the retained nucleosomes are still significantly enriched at loci of developmental importance, indicating that epigenetic marking in sperm is extensive and correlated with developmental regulators [34]. Mature sperm are highly abundant in small RNAs, including microRNAs (miRNAs), transfer RNA (tRNA)‐derived small RNAs (tsRNAs), and ribosomal RNA (rRNAs) [35, 36]. Most of small RNAs could be modulated by paternal environmental conditions and potentially delivered to the zygote at fertilization, where they can regulate early embryonic development and even affect long‐term health in offspring. Intriguingly, perturbations such as parental stress, malnutrition, infection, or advanced age have been associated with an increased incidence of neurodevelopmental disease in offspring through sperm small RNAs [27, 28, 37, 38, 39, 40].

In this study, we focused on whether the adverse effect of paternal circadian disruption can be transmitted to the subsequent generation via a sperm small RNA‐mediated intergenerational inheritance. Male C57BL/6 mice were exposed to a standard 12h‐light/12h‐dark cycle (LD) or constant light (LL) for 8 weeks and then mated with normal females. We conducted the behavioral tests in the offspring (F1). Since the male offspring have a significant difference in cognition but not females, we studied the hippocampal function in F1 males, and the transcriptional regulation of F0 sperm on early embryos. Mechanistically, we investigated the effect of LL on the small RNAs expression profile of F0 sperm, as well as the small RNAs expression in the sperm of men with irregular circadian rhythm. We used zygote injection and embryo transplantation model to clarify the potential epigenetic mediators contributing to the intergenerational cognitive abnormalities caused by circadian disruption. Additionally, we explored the chromatin accessibility in spermatogonial stem cells, and whether the neutralization of miR‐92a‐3p/miR‐25‐3p would cause phenotypic reversal of cognitive anomalies in LL offspring.

## 2 Results

### 2.1 Paternal Circadian Disruption Causes Cognitive Impairment in F1 Male, but not Female, Offspring

**FIGURE 1:**
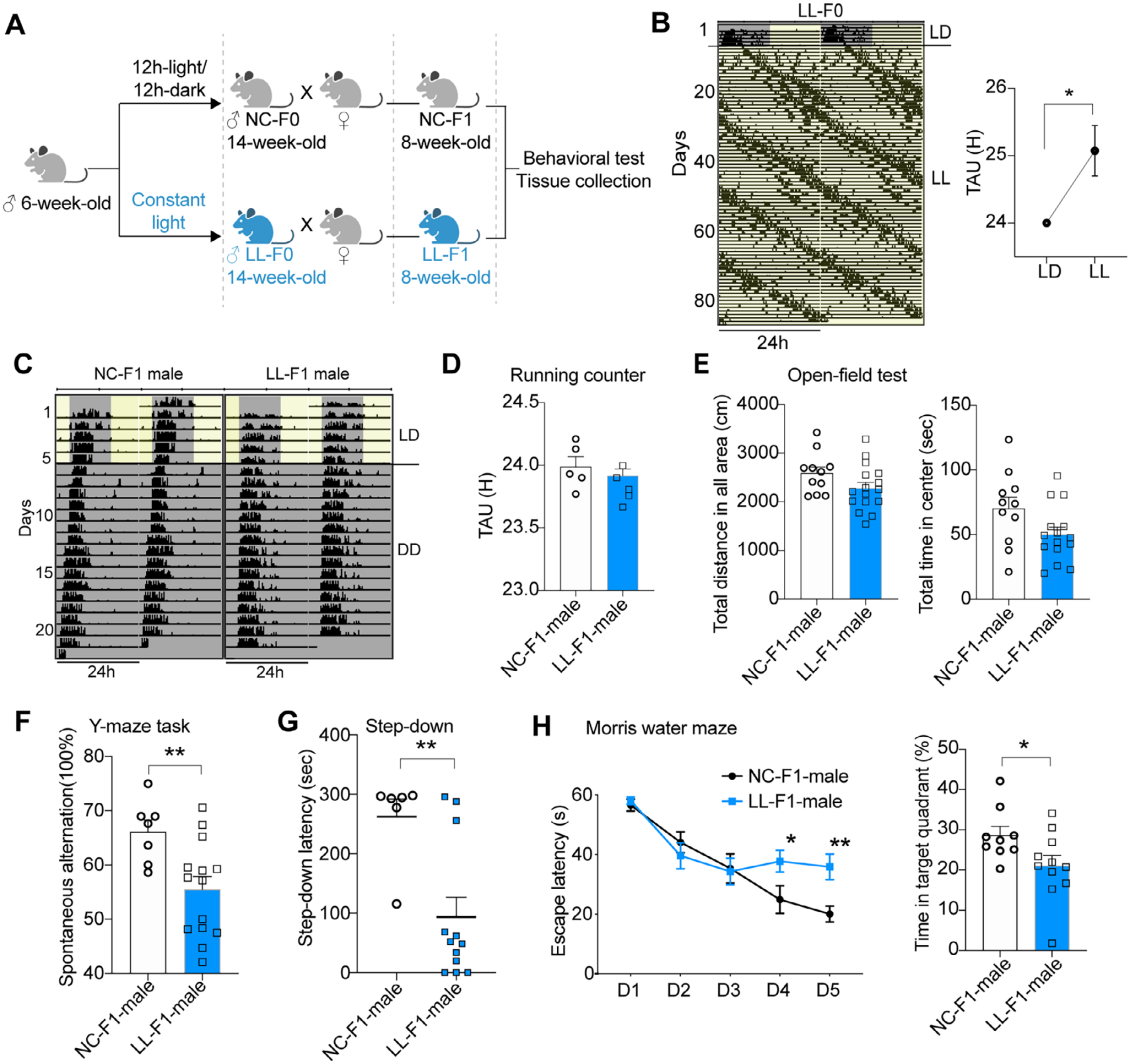
Paternal circadian disruption causes cognitive impairment in the F1 male offspring. (A) Schematic diagram. (B) Representative actograms of F0 male mice housed in a 12h:12 h light/dark cycle (LD) for 7 days, followed by 8 weeks in constant light conditions (LL). Data were displayed in a double‐plotted actogram. Free running period (TAU) was defined as the time interval between the onset of consecutive activity peaks, calculated from the actograms. Scale bar: 24 h. n_LD_ = 8, n_LL_ = 8 mice. (C) Representative actograms of NC‐F1 and LL‐F1 male mice housed in LD for 7 days, followed by 14 days in constant dark conditions (DD). Data were displayed in a double‐plotted actogram. (D) Quantification of free running periods for LL‐F1 male mice and control mice. n_NC‐F1‐male_ = 5, n_LL‐F1‐male_ = 5. (E) Anxiety‐like phenotype and locomotor activity of F1 mice was assessed using the open‐field test by measuring total distance traveled in the open field and time spent in center, respectively. n_NC‐F1‐male_ = 11, n_LL‐F1‐male_ = 15 mice. (F) Spatial working memory in male mice was calculated as a percentage of right alternations on the Y‐maze test. n_NC‐F1‐male_ = 7, n_LL‐F1‐male_ = 14 mice. (G) Long‐term memory analyzed 24 h after training session in the step‐down passive avoidance test. n_NC‐F1‐male_ = 6, n_LL‐F1‐male_ = 12 mice. (H) Time spent before reaching the hidden platform in the MWM, and time spent in the target quadrant during MWM test after the hidden platform was removed from the original location. n_NC‐F1‐male_ = 9, n_LL‐F1‐male_ = 11 mice. In all graphs, error bars represent mean ± SEM. For all behavioral assays, individual mice are plotted, and litter was treated as a random factor in mixed‐effects models to account for the non‐independence of siblings. All *p*‐values were obtained from mixed‐effects models unless otherwise indicated. The free‐running period was derived from onset‐to‐onset regression in ActogramJ. Data were analyzed by two‐tailed unpaired t test (B, D) between two groups. ^*^ *p* < 0.05, ^**^ *p* < 0.01.

To investigate the intergenerational effects of paternal circadian disruption, 6‐week‐old male C57BL/6 mice were randomly assigned to standard light–dark cycles (NC‐F0) or constant light (LL‐F0) for 8 weeks before mating with untreated females to generate NC‐F1 and LL‐F1 offspring (Figure 1A). Body weight and glucose tolerance did not differ between NC‐F0 and LL‐F0 males prior to mating (Figure S1A,B), although LL exposure lengthened the circadian period as expected (Figure 1B). Constant light markedly disrupted circadian organization in F0 males, as evidenced by flattened corticosterone rhythms, dampened and disordered clock‐gene oscillations across hypothalamus, liver, and testis, and blunted metabolic and behavioral rhythms in metabolic cage recordings (Data S1). These findings confirm that LL induces robust systemic circadian desynchronization prior to mating. Sperm motility, viability, and morphology were comparable between groups (Table S1 and Figure S1C), and fertility parameters also showed no significant differences (Table S2). Birth weight and post‐weaning growth of the offspring were similar between NC‐F1 and LL‐F1 litters (Figure S1D,E), and the free‐running period of NC‐F1 and LL‐F1 males did not differ (Figure 1C,D; Data S1).

We performed behavioral tests on NC‐F1 and LL‐F1 adult mice at 8 to 12 weeks old. In the open field test, there is no significant difference in the total distance in all area or total time in the center between NC‐F1 and LL‐F1 males, as well as females (Figure 1E; Figure S1F), suggesting the behavioral phenotypes were independent of locomotor activity. To investigate the learning and memory behavior, Y‐maze test, step‐down and Morris water maze test were carried out. In Y‐Maze test, we found impaired short‐term spatial memory in LL‐F1 male mice (Figure 1F). Long‐term memory analyzed 24 h after training session in the step‐down passive avoidance test, LL‐F1 male mice displayed less latency as compared to NC‐F1 (Figure 1G), indicating the impaired long‐term memory in LL‐F1 male mice. Similar results were found when spatial memory acquisition was assessed with the Morris water maze (MWM). LL‐F1 male mice spent less time in the target quadrant when compared to NC‐F1 males, showing impaired spatial working memory (Figure 1H). All behavioral parameters are provided in the raw dataset (Data S2). Interestingly, we found sex difference in the phenotypes of the offspring. Unlike males, LL‐F1 female mice showed no difference in the cognitive behavioral tests compared to NC‐F1 female mice (Figure S1F–I). These results indicate that paternal circadian disruption could cause significant behavioral change especially cognitive impairment in the F1 male offspring, but not F1 female offspring. Therefore, in this study, we mainly focused on the male offspring.

### 2.2 LL‐F1 Hippocampus Exhibits Impaired Synaptic Plasticity and Abnormal Expression of Genes and Proteins Involved in Neurotransmission

**FIGURE 2:**
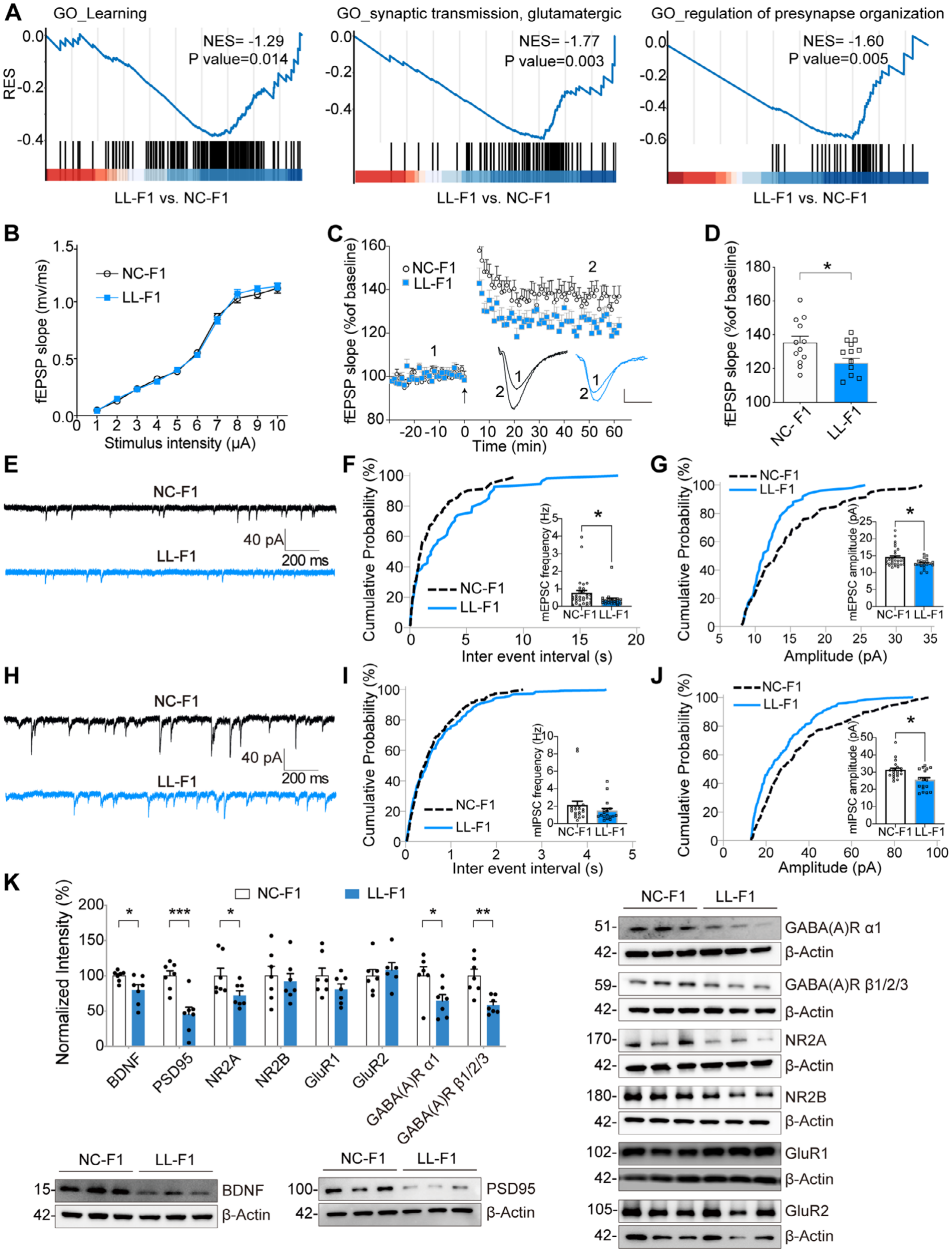
LL‐F1 hippocampus exhibits impaired synaptic and abnormal expression of genes and proteins involved in neurotransmission. (A) RNA‐seq was performed on F1 hippocampal tissue (*n* = 3 per group). GSEA graphs for learning, regulation of synaptic transmission, glutamatergic, and regulation of presynapse organization sets and their *p‐*value and normalized enrichment score (NES) are shown to illustrate the most interesting findings. A positive normalized enrichment score here indicates that genes related to a specific function show a trend to be overexpressed in LL‐F1; a negative normalized enrichment score indicates that genes related to a specific function show a trend to be under‐expressed in LL‐F1. (B) Similar amplitudes of fEPSP slopes. fEPSPs were recorded in the hippocampus with stimulation of the SC‐CA1 pathway at gradually increasing intensities. *n* = 12 slices from 3 mice for each group. (C) Impaired LTP at SC‐CA1 synapses in the hippocampus of LL‐F1 mice. Normalized fEPSP slopes were plotted every 1 min. Arrow denotes LTP induction. Shown on the right are representative traces taken before (1) and 50 min after tetanic stimulation (2). Scale bars: 10 ms, 0.2 mV. (D) Quantitative analysis of LTP level in (C). *n* = 12 slices from 3 mice for each group. (E) Representative traces of mEPSCs in CA1 pyramidal neurons from control and LL‐F1 male mice. Scale bar: 0.2 s, 40 pA. (F,G) Cumulative probability curve of mEPSC inter‐event intervals and histograms of mEPSC frequency (F) and amplitude (G). *n* = 30 neurons, 5 control mice; *n* = 21 neurons, 4 LL‐F1 male mice. (H) Representative traces of mIPSCs in CA1 pyramidal neurons from control and LL‐F1 male mice. Scale bar: 0.2 s, 40 pA. (I,J) Cumulative probability curve of mIPSC inter‐event intervals and histograms of mIPSC frequency (I) and amplitude (J). *n* = 20 neurons, 3 control mice; *n* = 17 neurons, 3 LL‐F1 male mice. (K) Relative protein levels in the mouse hippocampus assessed by Western blotting. Representative blots from three independent experiments with consistent results are shown. Quantification was performed on samples from n ≥ 4 mice per group. Protein band intensities were normalized to the loading control β‐actin, and values from control mice were set to 100%. Data were analyzed by two‐tailed unpaired t test (D,F,G,I,J,K), two‐way ANOVA followed by Fisher's *LSD* post hoc test (B,C). Kolmogorov‐Smirnov test estimated the cumulative probability. ^*^ *p* < 0.05, ^**^ *p* < 0.01, ^***^ *p* < 0.001.

Synaptic dysfunction is implicated in various neurological and psychiatric disorders. As no differences were detected in hippocampal levels of glutamate, glutamine, GABA, or other major neurotransmitters between NC‐F1 and LL‐F1 males (Figure S2A), we performed bulk RNA‐seq to assess transcriptional alterations. Using a screening threshold of nominal *p*‐value < 0.05 and |log2 fold change (FC)| > 1, hippocampal RNA‐seq identified a clear set of differentially expressed genes (DEGs) between groups, including a marked downregulation of *Fos*, an activity‐dependent immediate early gene, as shown in the volcano plot and heatmap (Data S3 and Figure S2B,C). Gene set enrichment analysis (GSEA) of hippocampi indicated that learning (NES = −1.29), synaptic transmission of glutamatergic (NES = −1.77) and regulation of presynapse organization (NES = −1.60) pathways were significantly down‐regulated in LL‐F1 male mice compared to the control group (Figure 2A).

We next examined synaptic transmission and plasticity at Schaffer collateral–CA1 (SC–CA1) synapses, a canonical hippocampal circuit involved in learning and memory. Long‐term potentiation (LTP) in the CA1 region was assessed as a widely used cellular correlate of learning and memory [41]. LTP was significantly impaired in LL‐F1 slices, as reflected by a reduced post‐tetanus fEPSP slope compared with NC‐F1 controls (Figure 2B–D). Whole‐cell recordings were then obtained from CA1 pyramidal neurons in acute hippocampal slices prepared from age‐matched NC‐F1 and LL‐F1 male mice (8–10 weeks old). Both the frequency and amplitude of miniature excitatory postsynaptic currents (mEPSCs) were significantly reduced in LL‐F1 males (Figure 2E–G). In contrast, the amplitude of miniature inhibitory postsynaptic currents (mIPSCs) was reduced without a change in frequency (Figure 2H–J). These findings indicate reduced excitatory synaptic input to CA1 pyramidal neurons together with altered inhibitory postsynaptic responses, consistent with disrupted hippocampal synaptic function in LL‐F1 male offspring.

We then examined proteins critical for synaptic transmission and plasticity, including brain‐derived neurotrophic factor (BDNF), postsynaptic density protein 95 (PSD95), GABAA receptor subunits (GABRA1, GABRB1, GABRB2, and GABRB3), N‐methyl‐D‐aspartate receptor subunits (NR2A and NR2B), and AMPA receptor subunits (GluR1 and GluR2). Compared with controls, protein levels of BDNF, PSD95, GABRA1, GABRB1–3, and NR2A were significantly reduced in adult LL‐F1 male mice (Figure 2K). Consistently, immunofluorescence analysis of the hippocampal CA1, CA3, and dentate gyrus regions showed reduced SYN1 and PSD95 signal intensity in LL‐F1 males (Figure S2D–F), further supporting impaired synaptic integrity at both pre‐ and postsynaptic sites. Together, these data indicate that paternal circadian disruption impairs hippocampal synaptic transmission and plasticity and is associated with reduced expression of synaptic plasticity‐related proteins in male offspring.

### 2.3 Sperm Small RNAs Contribute to Paternal Circadian Disruption‐Induced Cognitive Impairment

**FIGURE 3:**
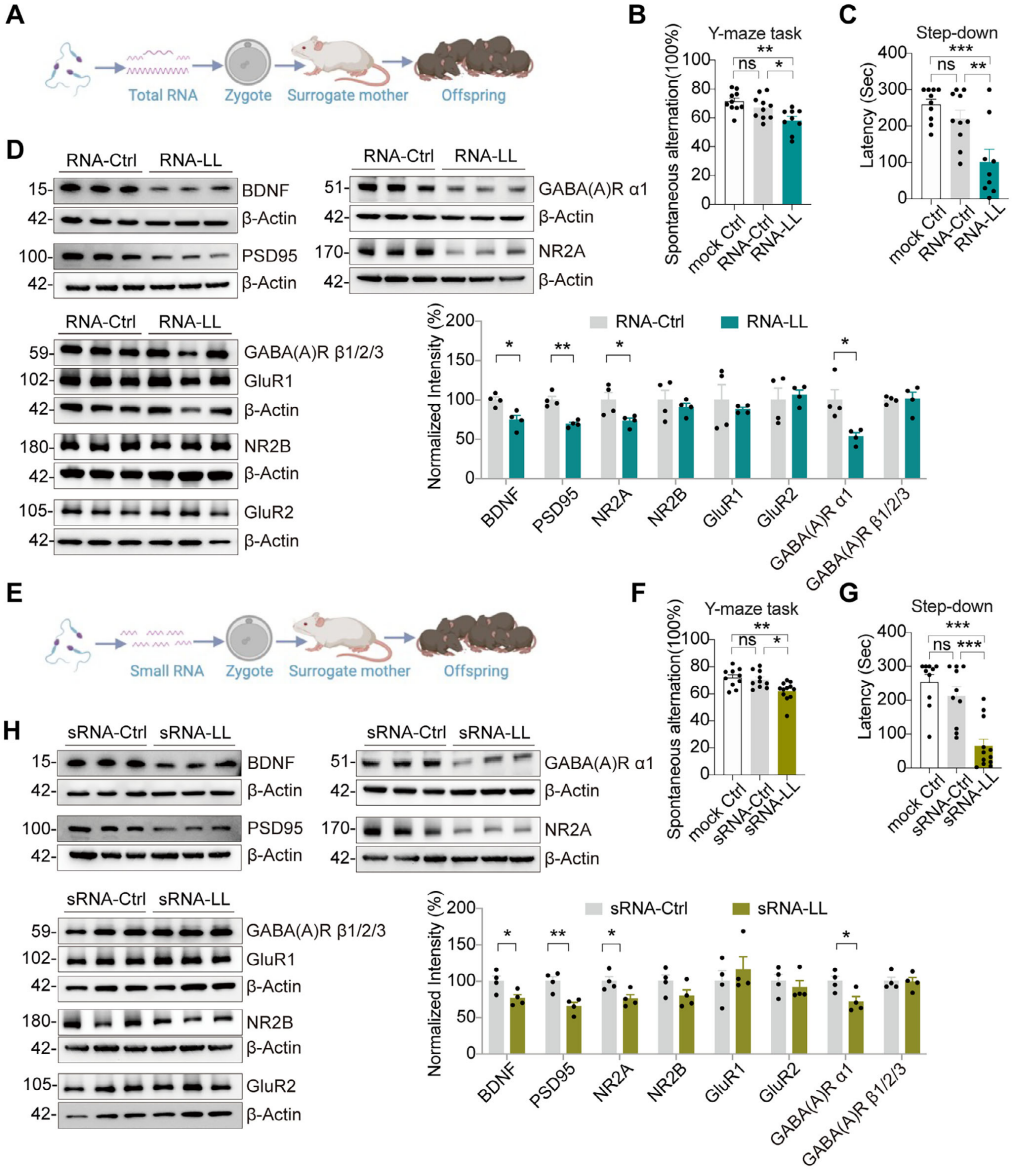
Sperm small RNAs contribute to paternal circadian disruption‐induced cognitive impairment. (A) Schematic timeline of IVF‐derived offspring generated from zygotes injected with sperm total RNA (RNA‐LL vs. RNA‐Ctrl / Mock Ctrl). The Mock Ctrl group received microinjection of DEPC‐treated water. (B) and (C) Behavioral performances in Y‐maze and Step‐down tests. n_mock Ctrl_ = 10, n_RNA‐Ctrl_ = 10, n_RNA‐LL_ = 9 mice. (D) and (H) Relative protein levels in the mouse hippocampus assessed by Western blotting. Representative blots from three independent experiments with consistent results are shown. Quantification was performed on samples from n ≥ 4 mice per group. Protein band intensities were normalized to the loading control β‐actin, and values from control mice were set to 100%. (E) Schematic timeline of IVF‐derived offspring generated from zygotes injected with sperm small RNAs (sRNA‐LL vs. sRNA‐Ctrl / Mock Ctrl). The Mock Ctrl group received microinjection of DEPC‐treated water. (F) and (G) Behavioral performances in Y‐maze and Step‐down tests. n_mock Ctrl_ = 10, n_sRNA‐Ctrl_ = 10, n_sRNA‐LL_ = 12 mice. Behavioral data (B,C,F,G) were analyzed using nested linear mixed‐effects models (Group as a fixed factor and Litter as a random factor), followed by Tukey post‐hoc comparisons. Western blot quantification (D,H) was analyzed using two‐tailed unpaired t test. ^*^ *p* < 0.05, ^**^ *p* < 0.01, ^***^ *p* < 0.001.

Previous studies have shown that sperm small RNA could potentially mediate the paternal effect on long‐term health in offspring. To evaluate whether sperm RNA of LL‐F0 related to the increased risk of cognition impairment in offspring, total RNAs from the sperm of NC‐F0 or LL‐F0 mice were purified and injected into normal zygotes, respectively (RNA injection was normalized to the amount of approximately 10 sperm). The embryos were then transferred into surrogate mothers to generate control F1 offspring (RNA‐Ctrl group) and LL‐F1 offspring (RNA‐LL group). Diethylpyrocarbonate (DEPC) water was injected into normal zygotes to generate offspring as a mock control (mock Ctrl group) (Figure 3A). We focused our behavioral analyses on male offspring because paternal LL exposure produced robust and reproducible cognitive impairments specifically in males, while female offspring showed no detectable deficits under the same testing conditions. The behavioral tests were conducted among the three groups. Compared with mock Ctrl group and RNA‐Ctrl group, the RNA‐LL group showed significant short‐term and long‐term memory impairment in Y‐maze task and Step‐down, which was similar to the changes in LL‐F1 group (Figure 3B,C). The protein levels of *BDNF*, *PSD95*, *NR2A* AND *GABRA1* were significantly decreased in hippocampi of RNA‐LL group (Figure 3D). Open field and elevated plus maze analysis confirmed that locomotor activity and anxiety‐like behavior were unchanged among groups, indicating that the observed cognitive deficits were not affected by movement or anxiety, which were comparable across all microinjection‐derived groups (Data S4).

Further, we purified small RNAs from the sperm of NC‐F0 or LL‐F0 mice and injected them into normal zygotes, respectively. The embryos were then transferred into surrogate mothers to generate control F1 offspring (sRNA‐Ctrl group) and LL‐F1 offspring (sRNA‐LL group). DEPC water was injected into normal zygotes to generate mock Ctrl group (Figure 3E). Compared with mock Ctrl group and sRNA‐Ctrl group, the sRNA‐LL group also showed significant short‐term and long‐term memory impairment in Y‐maze task and Step‐down, which was similar to the changes in LL‐F1 group and RNA‐LL group (Figure 3F,G). The protein levels of *BDNF*, *PSD95*, *NR2A* AND *GABRA1* were also significantly decreased in hippocampi of sRNA‐LL group (Figure 3H).

These results indicated that the RNAs of LL‐F0 sperm, especially small RNAs, probably mediate the cognitive behavioral alterations in the LL‐F1 group induced by paternal circadian rhythm disorders.

### 2.4 Circadian Disruption Increases Sperm miR‐92a‐3p/miR‐25‐3p, While Synthetic miR‐92a‐3p/miR‐25‐3p Injection Causes Cognitive Dysfunction

**FIGURE 4:**
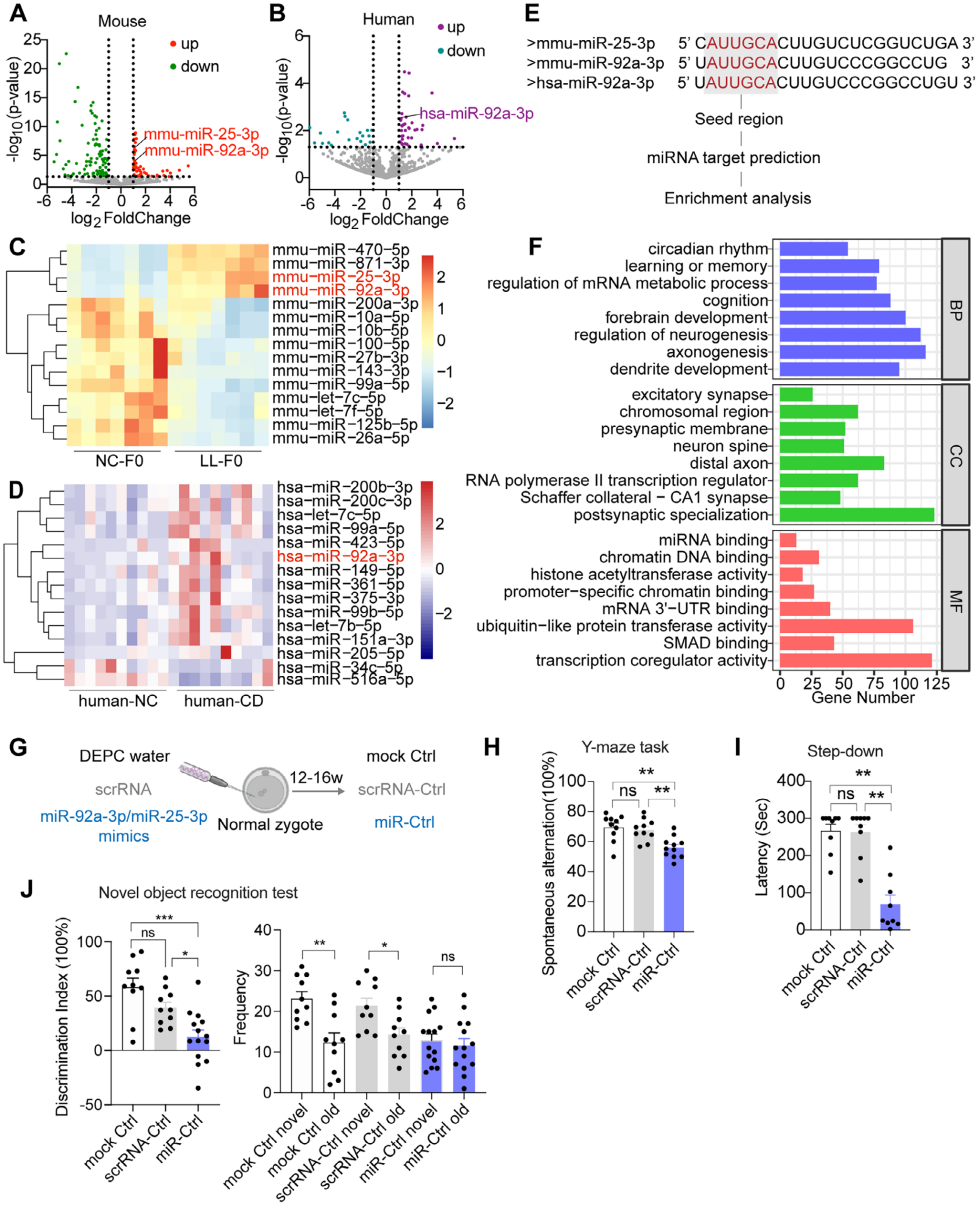
Circadian disruption increases sperm miR‐92a‐3p/miR‐25‐3p, while synthetic miR‐92a‐3p/miR‐25‐3p injection causes cognitive dysfunction. (A,C) Volcano plot and heatmap showing differentially expressed miRNAs in mouse sperm between LD‐F0 and LL‐F0 groups (*n* = 7 per group). (B,D) Volcano plot and heatmap showing differentially expressed miRNAs in human sperm between the human‐NC and human‐CD groups (*n* = 10 per group). (E) Mature sequences of miR‐92a family members and workflow of the conducted analysis. Red‐colored nucleotides indicate the shared seed sequence. (F) Functional enrichment analysis of predicted targets of miR‐92a‐3p and miR‐25‐3p. BP, biological process; MF, molecular function; CC, cellular component. (G) Zygotes injection scheme. Two specific miRNAs (miR‐92a‐3p and miR‐25‐3p) or scrambled negative control RNA (scrRNA) were injected into the cytoplasm of normally fertilized oocytes. Zygotes injected with miR‐92a‐3p/miR‐25‐3p or scrRNA were designated miR‐Ctrl and scrRNA‐Ctrl, respectively. The Mock Ctrl group received microinjection of DEPC‐treated water. Offspring derived from these injected zygotes were subjected to behavioral assessments at 12–16 weeks of age. (H, I) Behavioral performances in Y‐maze and Step‐down tests. n_mock Ctrl_ = 9–10, n_scrRNA‐Ctrl_ = 9–10, n_miR‐Ctrl_ = 9–11 mice. (J) Novel object recognition (NOR). Left: Discrimination index (DI) calculated as (time exploring the novel object − time exploring the familiar object) / total exploration time. Right: Exploration frequency toward the novel and familiar objects. n_mock Ctrl_ = 10, n_scrRNA‐Ctrl_ = 10, n_miR‐Ctrl_ = 14 mice. For the volcano plot, the x‐axis represents the log2 fold change, and the y‐axis represents the ‐log_10_ *p*‐value. Behavioral data were analyzed using nested linear mixed‐effects models (Group as a fixed factor and Litter as a random factor), followed by Tukey post‐hoc comparisons. ^*^ *p* < 0.05, ^**^ *p* < 0.01, ^***^ *p* < 0.001.

We performed small RNA sequencing on F0 sperm. Small RNA‐seq confirmed the expected miRNA length distributions in both mouse and human sperm samples (Data S5). Across the three annotated small‐RNA biotypes, miRNAs exhibited the clearest separation between groups in principal component analysis (PCA), and tsRNA and rsRNA changes were comparatively modest (Figure S3A and Data S6). For candidate discovery, miRNAs were screened using the criteria of nominal *p*‐value < 0.05 and |log2FC| > 1. In mouse sperm, paternal LL exposure induced broad changes in the miRNA landscape. Compared with NC‐F0, 140 miRNAs met these screening criteria in LL‐F0 sperm, including 48 upregulated and 92 downregulated miRNAs (Figure 4A). We also performed small RNA sequencing on sperm from men with normal circadian rhythm (human‐NC) or circadian desynchrony (human‐CD). In human sperm, the sequencing changes associated with circadian desynchrony were weaker and more heterogeneous than those observed in mice, with 38 upregulated and 21 downregulated miRNAs identified under the same screening criteria (Figure 4B).

To prioritize candidates, we focused on 18 upregulated miRNAs with baseMean > 100. Target prediction via multiMiR revealed functional enrichment in axonogenesis, synapse organization, and neuronal development (Data S7 and Figure S3B), suggesting that paternal circadian disruption induces a sperm miRNA profile targeting neurodevelopmental pathways. Notably, miR‐92a‐3p exhibited concordant upregulation across both mouse and human datasets, while miR‐25‐3p was significantly elevated in mouse sperm (Figure 4C,D). We confirmed these findings by RT–qPCR in LL‐F0 mouse sperm (nNC‐F0 = 6, nLL‐F0 = 6). Furthermore, RT–qPCR analysis of an independent human cohort validated the increase of miR‐92a‐3p in sperm from men with circadian desynchrony (Figure S3C), with participant characteristics summarized in Table S3. Among these, miR‐92a‐3p and miR‐25‐3p were selected for functional validation based on their robust enrichment scores and relatively high abundance.

Mechanistically, miR‐25‐3p shares the same seed sequence as miR‐92a‐3p (AUUGCA), suggesting potential cooperative regulation of common downstream genes (Figure 4E; Data S8). Functional enrichment analysis of predicted target genes implicated pathways relevant to brain development and synaptic function, including learning or memory, cognition, regulation of neurogenesis, dendrite development, excitatory synapse, presynaptic membrane, neuron spine, Schaffer collateral–CA1 synapse, postsynaptic specialization, miRNA binding, and mRNA 3′‐UTR binding (Figure 4F).

To test whether increased miR‐92a‐3p/miR‐25‐3p is functionally sufficient to affect offspring phenotypes, scrambled control RNA or synthetic miR‐92a‐3p/miR‐25‐3p mimics were injected into normal zygotes. Injected embryos were then transferred into surrogate mothers to generate scrRNA‐Ctrl and miR‐Ctrl offspring, respectively. DEPC‐treated water injection served as the mock Ctrl group (Figure 4G). Compared with mock Ctrl and scrRNA‐Ctrl offspring, miR‐Ctrl offspring showed impaired spatial memory and long‐term memory in the Y‐maze and step‐down tests, resembling the phenotypes observed in LL‐F1, RNA‐LL, and sRNA‐LL groups (Figure 4H,I). In the novel object recognition test, miRNA mimics injected offspring also exhibited a reduced discrimination index and lower preference for the novel object (Figure 4J), further supporting impaired recognition memory in the miR‐Ctrl group. Together, these results support miR‐92a‐3p and miR‐25‐3p as functionally important candidate mediators of the intergenerational cognitive alterations associated with paternal circadian disruption.

### 2.5 Circadian Rhythm Disruption Reprograms Chromatin Accessibility and miRNA Expression in Undifferentiated SSCs

**FIGURE 5:**
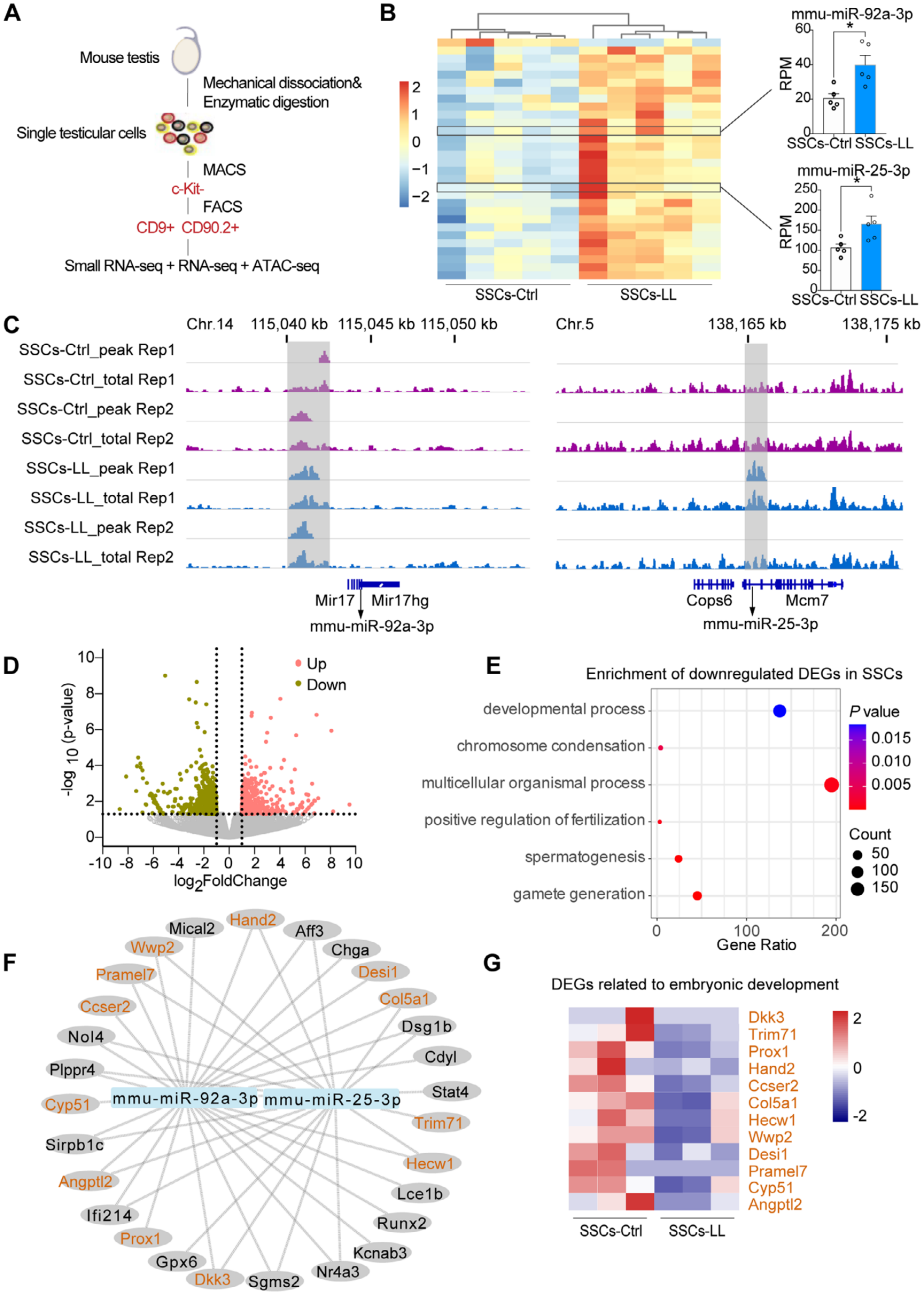
Circadian rhythm disruption reprograms chromatin accessibility and miRNA expression in undifferentiated SSCs. (A) Experimental workflow for spermatogonial stem cells isolation from mouse testes and subsequent multi‐omics profiling. MACS, magnetic‐activated cell sorting; FACS, fluorescence‐activated cell sorting. (B) Heatmap of the top 30 differentially expressed miRNAs and normalized expression (RPM) of miR‐92a‐3p and miR‐25‐3p in SSCs‐LL versus SSCs‐Ctrl (*n* = 5 per group). (C) ATAC‐seq signal tracks at the miR‐92a‐3p locus on chromosome 14 and the miR‐25‐3p locus on chromosome 5. Single lane height is standardized across samples (*n* = 2 per group). (D) Volcano plot of RNA‐seq compared with SSCs‐LL and SSCs‐Ctrl group (*n* = 3 per group). (E) Gene Ontology (GO) enrichment analysis of the downregulated genes in SSCs. (F) miRNA–mRNA regulatory network in SSCs. Blue squares represent miRNAs, and gray circles represent downregulated DEGs targeted by miR‐92a‐3p/miR‐25‐3p (SSCs‐LL vs. SSCs‐Ctrl). miRNA–target relationships were obtained using the R package multiMiR. (G) Heatmap showing the expression patterns of the DEGs in (F) that are associated with embryonic development. Data were analyzed by two‐tailed unpaired t test (B). ^∗^ *p* < 0.05.

To examine whether circadian disruption alters the earliest stages of the male germline, we isolated undifferentiated spermatogonial stem cells (SSCs) from NC‐F0 and LL‐F0 testes. CD117^+^ differentiating spermatogonia were depleted by MACS, followed by FACS purification of CD9^+^CD90.2^+^ SSCs, a well‐established population with self‐renewal and spermatogenic potential (Figure 5A). These purified cells were subjected to small RNA‐seq, ATAC‐seq, and RNA‐seq to capture early molecular consequences of LL exposure. Small RNA‐seq revealed high‐quality libraries dominated by miRNAs and piRNAs (Figure S4). We applied RPM (reads per million reads) to quantify the miRNA expression. The sequencing and mapping information are shown in Data S9. Notably, miR‐92a‐3p and miR‐25‐3p were upregulated in SSCs from LL‐F0 males (Figure 5B).

Two biological replicates (rep 1–2) were prepared for each sample (SSCs‐Ctrl and SSCs‐LL) to analyze chromatin accessibility by ATAC‐seq. TSS‐heat map analysis revealed that there were no significant differences between the SSCs‐Ctrl and SSCs‐LL groups (Figure S5), indicating that LL exposure did not drastically change chromatin accessibility. miR‐92a‐3p is a part of the miR‐17‐92 gene cluster, which is located within the *Mir17hg* gene in the chromosome 14qC1 region. miR‐25‐3p is located within the miR‐106b‐25 gene cluster on chromosome 5qC3.3 and resides in the protein‐coding gene *Mcm7*. ATAC‐seq track analysis observed increased *Mir17hg* signal in the SSCs‐LL group compared to the control cells, and a similar pattern was also found at the *Mcm7* locus (Figure 5C). These observations indicate that circadian disruption can remodel chromatin landscapes at specific regulatory regions in SSCs.

RNA‐seq further revealed extensive transcriptional changes in SSCs after LL exposure, including 447 upregulated and 758 downregulated genes (Figure 5D). Gene Ontology analysis showed that the downregulated genes were mainly enriched in developmental process, chromosome condensation, and gamete generation (Figure 5E). Integration of the miRNA and mRNA datasets identified a set of downregulated genes predicted to be targeted by miR‐92a‐3p and miR‐25‐3p (Figure 5F), and several of these genes are linked to embryonic development, including *Cyp51* (Figure 5G). Together, these data show that circadian disruption is already associated with altered miRNA expression, chromatin changes, and developmental gene dysregulation in undifferentiated SSCs. Although these SSC data are not intended as direct evidence of inheritance, they support the idea that the molecular abnormalities detected later in sperm and embryos may originate from an earlier germline state.

### 2.6 miR‐92a‐3p/miR‐25‐3p Overexpression Recapitulates Circadian Disruption‐Induced Transcriptomic Changes In Embryos, While Their Antisense Inhibition In Zygotes Rescues LL‐F1 Abnormalities

**FIGURE 6:**
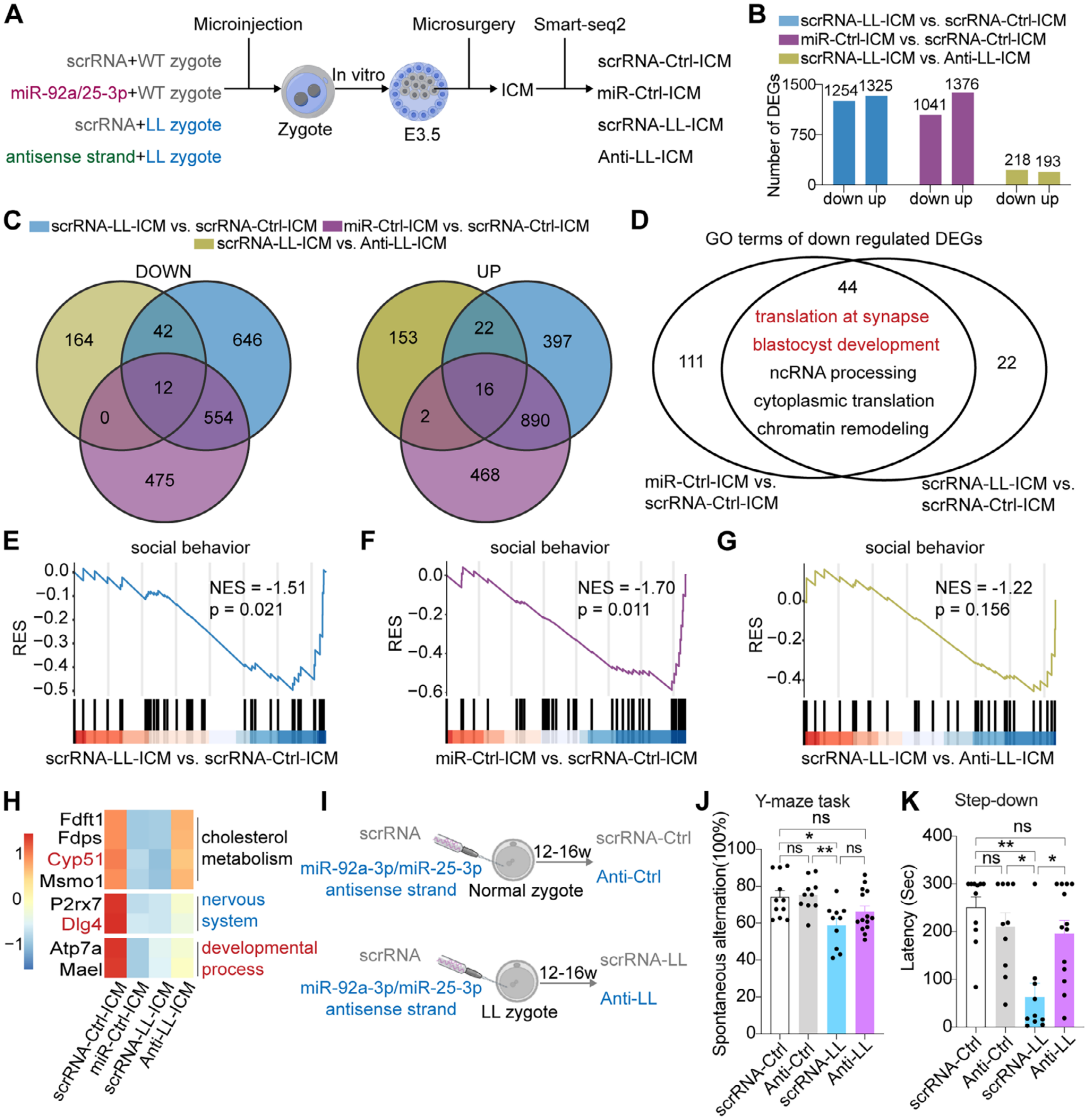
miR‐92a‐3p/miR‐25‐3p overexpression recapitulates circadian disruption‐induced transcriptomic changes in embryos, while their antisense inhibition in zygotes rescues LL‐F1 abnormalities. (A) Schematic diagram of ICM isolation and grouping. E3.5 mouse blastocysts were subjected to microsurgical isolation of the inner cell mass (ICM), which was subsequently used for Smart‐seq2 transcriptome profiling. Sample sizes: (n_scrRNA‐Ctrl‐ICM_ = 3, n_miR‐Ctrl‐ICM_ = 6, n_scrRNA‐LL‐ICM_ = 5, n_Anti‐LL‐ICM_ = 6). (B) Number of upregulated and downregulated DEGs in the indicated comparisons. (C) Venn diagram showing the overlap of down‐ or upregulated DEGs among scrRNA‐LL‐ICM vs. scrRNA‐Ctrl‐ICM, miR‐Ctrl‐ICM vs. scrRNA‐Ctrl‐ICM, and scrRNA‐LL‐ICM vs. Anti‐LL‐ICM. (D) Venn diagram shows the specific and common significantly enriched GO terms between the comparison groups. (E–G) GSEA‐based GO enrichment analysis. Gene enrichment plots for the social behavior pathway in scrRNA‐LL‐ICM vs. scrRNA‐Ctrl‐ICM, miR‐Ctrl‐ICM vs. scrRNA‐Ctrl‐ICM, and scrRNA‐LL‐ICM vs. Anti‐LL‐ICM. (H) Heatmap showing pseudobulked group‐level expression of embryonic development related genes across ICM samples (same sample numbers as listed in A). (I) Zygotes injection scheme. Control zygotes injected with scrRNA or miR‐92a‐3p/miR‐25‐3p antisense strands were “scrRNA‐Ctrl” and “Anti‐Ctrl” respectively. LL zygotes from LL male mice injected with scrRNA or miR‐92a‐3p/miR‐25‐3p antisense strands, were “scrRNA‐LL” and “Anti‐LL” respectively. Offspring were assessed behaviorally at 12–16 weeks of age. (J) and (K) Behavioral performances in Y‐maze and Step‐down tests. n_scrRNA‐Ctrl_ = 11, n_anti‐Ctrl_ = 10, n_scrRNA‐LL_ = 10, n_anti‐LL_ = 13–14 mice. Behavioral data (J,K) were analyzed using nested linear mixed‐effects models (Group as a fixed factor and Litter as a random factor), followed by Tukey post‐hoc comparisons. ^*^ *p* < 0.05, ^**^ *p* < 0.01, ^***^ *p* < 0.001. GSEA, gene set enrichment analysis; GO, gene ontology; NES, normalized enrichment score.

To determine whether elevated miR‐92a‐3p/miR‐25‐3p levels alter early embryonic development and whether inhibiting these miRNAs could mitigate LL‐induced abnormalities, we performed targeted zygotic microinjection. Control zygotes were injected with scrambled RNA (scrRNA) or synthetic miR‐92a‐3p/miR‐25‐3p mimics, whereas LL‐derived zygotes were injected with either scrRNA or the corresponding antisense inhibitors (antagomiRs). Embryos were cultured to the blastocyst stage (E3.5), and inner cell masses (ICMs) were isolated for Smart‐seq2 analysis, generating the scrRNA‐Ctrl‐ICM (*n* = 3), miR‐Ctrl‐ICM (*n* = 6), scrRNA‐LL‐ICM (*n* = 5), and Anti‐LL‐ICM (*n* = 6) groups (Figure 6A).

Differential gene expression analysis of the ICM transcriptomes revealed substantial changes across experimental groups. Compared with the scrRNA‐Ctrl‐ICM group, the scrRNA‐LL‐ICM group exhibited 1,254 downregulated genes and 1,325 upregulated genes. In the miR‐Ctrl‐ICM group, compared with the scrRNA‐Ctrl‐ICM group, a total of 1,041 genes were downregulated and 1,376 genes were upregulated. Interestingly, a comparison between the scrRNA‐LL‐ICM and Anti‐LL‐ICM groups identified 218 downregulated genes and 193 upregulated genes, indicating that miRNA inhibition can partial reverse the transcriptomic changes caused by LL (Figure 6B). A Chi‐square test (*p* = 4.5 × 10^−^^1^ ^2^) confirmed that the rescue overlap significantly exceeded random expectation. Venn diagram analysis of DEGs among the three pairwise comparisons revealed substantial overlap between the miR‐Ctrl‐ICM vs. scrRNA‐Ctrl‐ICM and scrRNA‐LL‐ICM vs. scrRNA‐Ctrl‐ICM groups. This indicated that overexpression of miR‐92a‐3p and miR‐25‐3p in control zygotes induces transcriptomic changes that mimic those observed in LL zygotes. Furthermore, following antagomiR injection, a subset of the DEGs identified in the LL group exhibited reversed expression patterns, suggesting a partial rescue of the LL‐associated gene expression profile (Figure 6C).

GO enrichment analysis of downregulated DEGs also revealed a substantial overlap between the miR‐Ctrl‐ICM vs. scrRNA‐Ctrl‐ICM and scrRNA‐LL‐ICM vs. scrRNA‐Ctrl‐ICM comparisons. A total of 44 commonly enriched pathways were identified in both groups. These included key biological processes such as translation at synapse, blastocyst development, ncRNA processing, cytoplasmic translation, and chromatin remodeling, suggesting that miR‐92a‐3p and miR‐25‐3p overexpression recapitulates LL‐associated impairments in early embryonic gene regulation (Figure 6D). Gene Set Enrichment Analysis (GSEA) demonstrated that antagomiR injection partially restored the expression of pathways associated with social behavior, which were significantly downregulated in the scrRNA‐LL‐ICM and miR‐Ctrl‐ICM group, indicating a potential corrective effect of miRNA inhibition on LL‐induced transcriptomic dysregulation (Figure 6E–G). Heatmap showed that several genes closely involved in neurodevelopment were downregulated following both LL exposure and miR‐92a‐3p/miR‐25‐3p overexpression, while their expression levels were restored after antagomiR injection, indicating a rescue effect at the transcriptomic level. Notably, *Cyp51*, which was also found to be significantly downregulated in SSCs of LL‐exposed mice, exhibited a similar downregulation pattern in early embryos. In addition, Dlg4, which encodes the postsynaptic density protein PSD95, was among the downregulated genes and is consistent with the reduced PSD95 expression observed in the adult hippocampus of LL‐F1 male offspring, highlighting a potential link between early embryonic gene regulation and long‐term synaptic dysfunction (Figure 6H). Luciferase reporter assays further confirmed that miR‐92a‐3p and miR‐25‐3p directly bound the 3′UTRs of *Cyp51* and *Dlg4*, respectively, because both miRNAs significantly reduced luciferase activity from the wild‐type reporters but not from the corresponding seed‐mutant constructs (Figure S6A). To further assess the physiological relevance of these interactions in early embryos, we performed a dose‐titration experiment in zygotes injected with synthetic miR‐92a‐3p/miR‐25‐3p mimics and quantified CYP51 protein levels at the early morula stage by immunofluorescence. We tested 0.2, 1, and 2 ng/µL mimics, corresponding approximately to 1‐, 5‐, and 10‐sperm equivalents, respectively. Even the lowest dose was sufficient to reduce CYP51 signal relative to the scramble control (Figure S6B,C), indicating that repression of this target can occur at an approximately 1‐sperm‐equivalent dose.

To assess the functional impact of miR‐92a‐3p and miR‐25‐3p on offspring behavior, we performed microinjection into zygote followed by embryo transfer. Control zygotes were injected with either scrRNA or antisense inhibitors targeting miR‐92a‐3p and miR‐25‐3p (Anti‐Ctrl), while LL zygotes were injected with either scrRNA (scrRNA‐LL) or antagomir (Anti‐LL). Embryos were transferred into surrogate females, and behavioral testing was conducted in adult offspring aged 12–16 weeks. In the Y‐maze, scrRNA‐LL offspring showed significantly reduced alternation relative to scrRNA‐Ctrl (*p* = 0.0028), whereas Anti‐LL offspring displayed a rightward shift toward control performance. Although the improvement did not reach significance compared with scrRNA‐LL, Anti‐LL mice did not differ significantly from scrRNA‐Ctrl (*p* = 0.0855), indicating partial normalization of the distribution. Consistently, the step‐down test confirmed recovery of long‐term memory in Anti‐LL group. Together, these findings demonstrate that neutralization of miR‐92a‐3p and miR‐25‐3p partially rescues LL‐induced transcriptomic and neurocognitive abnormalities (Figure 6I–K).

### 2.7 miR‐92a‐3p/miR‐25‐3p Overexpression Disrupts Early Embryonic Transcriptomes in a Sex‐Specific Manner

**FIGURE 7:**
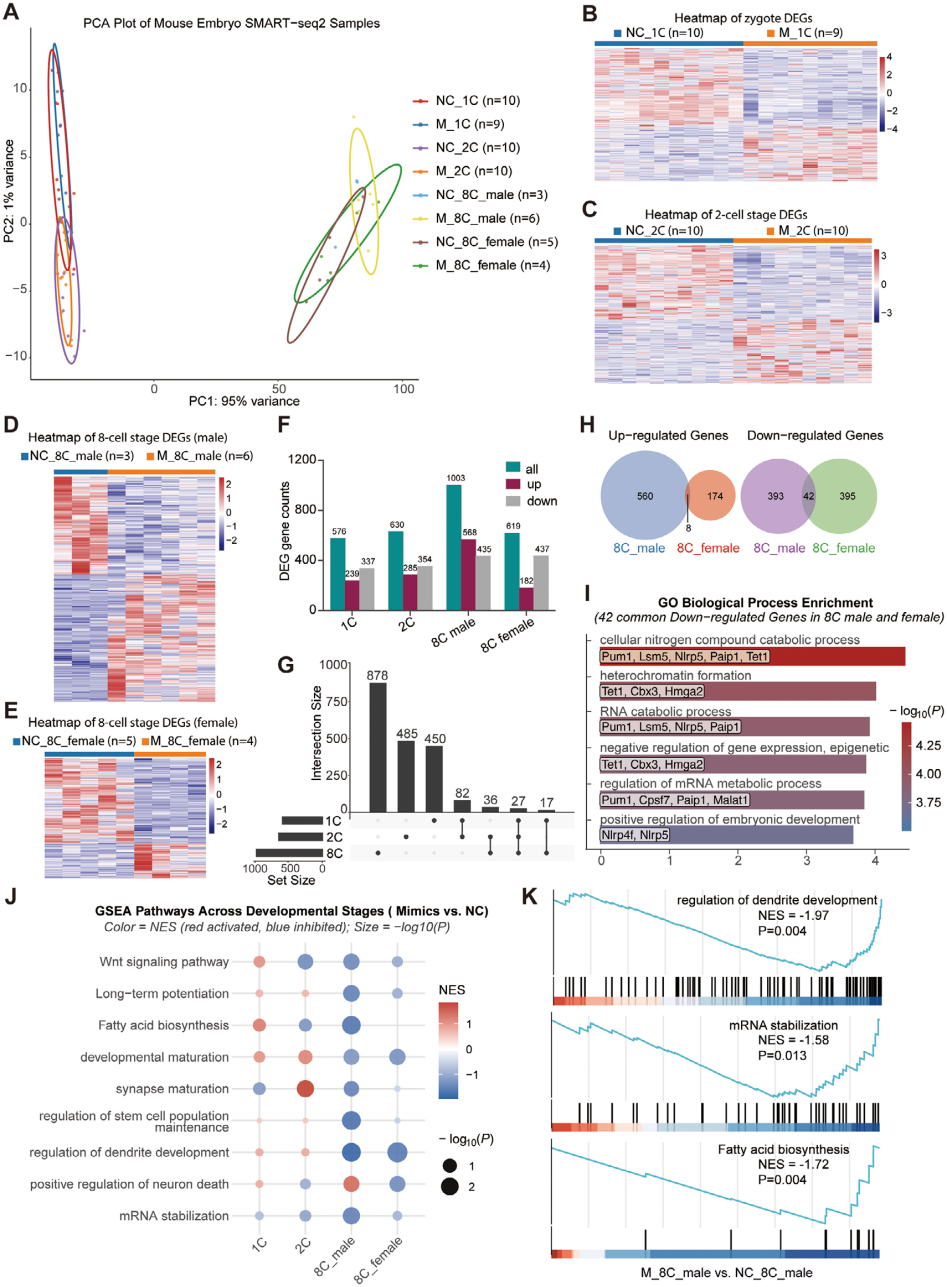
Single‐embryo Smart‐seq2 transcriptomic profiling of 1C, 2C, and 8C embryos following miR‐92a‐3p/miR‐25‐3p microinjection. Sample sizes are indicated within each panel. (A) Principal component analysis (PCA) of all Smart‐seq2 samples across the 1C, 2C, and 8C stages following microinjection of miR‐92a‐3p/miR‐25‐3p mimics (M) or scrambled RNA controls (NC). (B) Heatmap of DEGs in zygotes (1C) collected 2 h after microinjection. (C) Heatmap of DEGs in 2‐cell embryos. (D) Heatmap of DEGs in 8‐cell male embryos. (E) Heatmap of DEGs in 8‐cell female embryos. (F) Number of upregulated and downregulated DEGs across developmental stages. (G) Upset plot showing shared and stage‐specific DEGs among 1C, 2C, 8C_male, and 8C_female embryos. (H) Venn diagrams showing overlap of upregulated and downregulated DEGs between 8C_male and 8C_female embryos. (I) GO enrichment of the 42 commonly downregulated genes shared between 8C_male and 8C_female embryos, with corresponding genes shown for major pathways. (J) Bubble plot summarizing GSEA results across the four developmental stages (1C, 2C, 8C_male, 8C_female). Pathways related to embryonic development, neuronal differentiation, mRNA stability, and fatty acid metabolism are displayed; bubble size reflects ‐log_10_ (*P*); red indicates upregulation in the mimics group and blue indicates downregulation. (K) Representative GSEA enrichment plots (M_8C_male vs. NC_8C_male) for three key pathways shown in (J). GO and GSEA analyses were performed with M vs. NC comparisons for each embryonic stage.

Single‐embryo Smart‐seq2 revealed that sex could be reliably distinguished at the 8‐cell stage based on Y‐linked gene expression, and subsequent analyses were therefore stratified by sex at this stage. All subsequent analyses therefore compared mimics‐injected (M) versus scrambled controls (NC) within each developmental stage. Principal component analysis showed that 1C and 2C embryos clustered together, whereas 8C embryos separated strongly along PC1, which accounted for 95% of total variance, indicating a major transcriptomic divergence at this stage (Figure 7A). Heatmaps of DEGs across 1C, 2C, 8C male, and 8C female embryos confirmed high within‐group uniformity and clear between‐group segregation, demonstrating robust stage‐specific transcriptional responses to miRNA overexpression (Figure 7B–E).

Using nominal *p* < 0.05 and |log2FC| > 1 as thresholds, we identified 576 DEGs at the 1C stage (239 up, 337 down), 630 at 2C (285 up, 354 down), 1,003 in 8C male embryos (568 up, 435 down), and 619 in 8C female embryos (182 up, 437 down). UpSet analysis demonstrated limited overlap between early stages, 1C and 2C shared 82 DEGs, and 2C and 8C shared 36, whereas 878 DEGs were uniquely altered at the 8C stage (Figure 7F,G). Consequently, we focused on the 8C stage, where male and female embryos shared 8 commonly upregulated and 42 commonly downregulated genes (Figure 7H). GO analysis of these 42 shared downregulated DEGs revealed enrichment for pathways related to embryonic development, RNA catabolic processes, and negative regulation of gene expression, epigenetic regulation. Notably, these included Tet1, a core epigenetic regulator, and Nlrp5, a maternal‐effect gene essential for early embryogenesis, underscoring the functional relevance of miRNA‐driven suppression at 8C stage (Figure 7I).

To further dissect pathway‐level alterations across developmental stages, we performed GSEA. In 8C male embryos, multiple pathways important for neurodevelopment and cellular maturation, including Wnt signaling, long‐term potentiation, fatty acid biosynthesis, developmental maturation, synapse maturation, regulation of dendrite development, and mRNA stabilization, were significantly downregulated (NES < −1, *p* < 0.05), while “positive regulation of neuron death” was significantly upregulated (NES > 1, *p* < 0.05). Notably, these pathways showed minimal alteration at the 1C stage and remained largely unchanged at 2C, except for a significant downregulation of Wnt signaling, indicating progressive divergence culminating at 8C. Intriguingly, 8C female embryos exhibited an opposite response in “positive regulation of neuron death,” which was significantly downregulated rather than upregulated. Among the eight pathways suppressed in 8C males, two (developmental maturation and regulation of dendrite development) were also downregulated in females, five showed no significant change, and one (fatty acid biosynthesis) was not detected (Figure 7J). Representative enrichment plots for key pathways illustrate these transcriptional shifts (Figure 7K). Together, these data demonstrate that miR‐92a‐3p/miR‐25‐3p overexpression disrupts early embryonic gene regulatory programs in a manner that is both stage dependent and sexually dimorphic, with the strongest effects emerging at the 8‐cell stage.

## 3 Discussion

In this study, we used a mouse model of circadian disruption through constant light exposure in fathers and demonstrated that paternal circadian dysregulation impairs cognitive behavioral phenotypes in a sex‐dependent manner. Our discoveries underscore the role of spermatozoa in carrying transgenerational epigenetic inheritance and demonstrate that circadian rhythm disorder‐induced cognitive impairment can be transmitted through the male germline, mediated by specific miRNAs.

Our previous work also revealed a sexually dimorphic effect of gamete‐embryonic origin disorders on offspring, with male progeny exhibiting significant cognitive impairment or adverse glucose metabolism compared to females [42, 43, 44], consistent with emerging evidence that early developmental responses to environmental perturbations are sexual dimorphism [45]. Our embryo transcriptomic data further support this view by revealing distinct gene and pathway alterations between male and female 8‐cell embryos, indicating that paternal epigenetic signals interface with embryonic regulatory programs in a sex‐specific manner. These results suggest that different environmental exposures can induce distinct sex‐specific effects, probably mediated through sex hormone–dependent regulation of hippocampal plasticity [46, 47] and X/Y‐linked transcriptional [48] and epigenetic asymmetries established during preimplantation development [49, 50]. In parallel, male embryos show heightened sensitivity to paternal environmental signals, as evidenced across multiple paternal models [30, 51]. These convergent mechanisms provide a coherent explanation for the male‐biased cognitive and synaptic deficits observed in our study.

Previous studies have shown that mouse sperm tsRNAs are implicated in the acquired inheritance of metabolic disorders, while miRNAs are involved in the transmission of trauma or depression effects [25, 27, 30, 52]. Our findings, together with emerging evidence on paternal epigenetic inheritance, highlight the complex interplay between environmental stressors and germline epigenetic reprogramming. In our circadian disruption model, miRNAs emerged as key candidate mediators of offspring cognitive phenotypes, potentially acting by directly altering embryonic gene expression. We focused mechanistically on miR‐92a‐3p and miR‐25‐3p because they were conserved in both species and experimentally tractable for functional perturbation, while acknowledging that other small RNA classes or RNA modifications are also likely to contribute to the observed phenotypes [53, 54].

We further examined c‐Kit^−^CD9^+^CD90.2^+^ spermatogonial stem cells (SSCs) in mouse testes and found the trends of miR‐92‐3p and miR‐25‐3p expression were consistent with those in mature spermatozoa from circadian disrupted males. RNA‐seq analysis in these SSCs revealed that paternal circadian misalignment alters the expression of genes involved in embryonic development, whereas ATAC‐seq indicated increased chromatin accessibility at loci encoding the miR‐17‐92 and miR‐106b‐25 clusters, which may underlie the elevated miR‐92a‐3p/miR‐25‐3p expression. Our SSC analysis was designed to be exploratory, not to demonstrate the direct inheritance of SSC‐derived miRNAs, but to determine where circadian rhythm disruption first disrupts the germline regulatory environment. It is also important to note that environmental circadian disruption can produce heterogeneous reproductive or intergenerational outcomes depending on the specific model used. For example, paternal night‐restricted feeding disrupts circadian rhythms and affects offspring metabolic health via predominantly nongerm cell factors [55]. Likewise, studies emphasizing epididymal vesicle mediated small RNA remodeling show that post‐testicular contributions can dominate in shaping the mature sperm RNA payload and its impact on embryogenesis [56, 57, 58]. These considerations highlight that different environmental disturbances can affect different patterns in the germline regulatory network, which may explain variation in different studies.

Functionally, microinjection of miR‐92a‐3p/miR‐25‐3p mimics into wild‐type zygotes recapitulated the cognitive and molecular phenotypes of embryos derived from LL‐exposed father, whereas antisense inhibition partially restored developmental gene expression and cognitive performance. Our findings, along with emerging evidence, suggest that sperm‐derived RNAs play a crucial role in regulating embryogenesis and zygotic genome activation [51, 57, 59]. Importantly, the restoration of and *Cyp51* and *Dlg4* expression in antagomir injected embryos suggests that miRNA‐associated repression of genes may form a mechanistic link between early embryogenesis and adult cognitive outcomes.

Several points warrant more detailed discussion. First, evidence suggests that both seminal plasma (SP) and sperm contribute multiple factors that shape embryogenesis. Semen also plays role in driving early embryonic development, and describes how paternal factors, such as SP, sperm centriole, sperm proteins, sperm RNA, sperm DNA, and its integrity, together with epigenetics, may influence the female reproductive tract and post‐fertilization events [60]. Trigg et al. demonstrated that embryos fertilized by sperm without epididymal miRNAs display altered gene expression rescued by microinjection of epididymal miRNAs, providing evidence that soma‐to‐germline transferred miRNAs can regulate embryogenesis [58]. And our observation aligns with previous findings that elevated paternal glucocorticoid exposure can modulate sperm small RNAs and affect offspring neurodevelopment [61]. Although our primary focus here is on sperm small RNAs, we explicitly acknowledge that seminal components, including corticosterone and extracellular vesicles, may contribute to the observed molecular signatures. In our study, we found that miR‐92‐3p and miR‐25‐3p were more significantly differential in mature sperm than in SSCs, which may be caused by the change of miRNA expression profile in the epididymal microenvironment induced by circadian disruption. Second, there remains a lack of evidence linking paternal circadian disruption to offspring cognitive abnormalities in human studies, which needs to be validated in prospective cohort studies. At last, human sperm RNA profiles exhibit greater variability than those in controlled mouse experiments, reflecting differences in lifestyle, environment and genetic background. Therefore, our human results should be seen as translationally supportive rather than clinically diagnostic. Future studies will be essential to further clarify the contribution of circadian disruption to human sperm miRNA variation and predictive value for offspring outcomes.

## 4 Methods

### 4.1 Mouse Housing and Circadian Rhythm Recording

All experimental procedures were approved by the Institutional Animal Care and Use Committees at the Shanghai Model Organisms Center, and the ethical approval number is 2018‐002. C57BL/6 mice were housed at a constant temperature and humidity with ad libitum access to standard rodent diet, under the standard 12h‐light/12h‐dark cycles (LD, ∼150 lux white ambient illumination). In this study we used real clock time (h), because LL eliminates the external Zeitgeber. LD mice were maintained on a 12:12 light–dark cycle (lights on at 08:00; lights off at 20:00). The LL‐F0 male mice were exposed to constant light for eight weeks, from 6‐week‐old to 14‐week‐old. Locomotor activities were assessed using a customized wheel‐running system. Then F0 males were mated with normal circadian F0 females to breed F1 offspring. Mouse breeding was conducted in the standard mouse housing suites. To ensure equal nutrition, we normalized all litter sizes to 6–8 pups at postnatal day 3.

### 4.2 Behavioral Tests

Mice were handled by test performers for 3 days before any behavioral test. All the behavior tests were carried out between 12pm and 5pm. On the test day, mice were acclimated to the testing room for 1 h before testing. Unless otherwise noted, the apparatus used was cleaned thoroughly with 70% ethanol to avoid any olfactory cue bias after each session. Behavioral tests were performed on F1 mice aged 8 to 12 weeks old. We used offspring from 4 to 7 different litters per group and randomly selected 1–3 mice from each litter for testing.

### 4.3 Open‐Field Test

For the open‐field test, which is usually used in studies of the neurobiological basis of locomotor activity and anxiety, mice were placed in a chamber (42 × 42 × 30 cm) and movement was monitored for 15 min using an overhead camera and EthoVision XT 13.0 video‐tracking software (Noldus, Netherlands). The center 19 × 19 cm region was artificially defined as the center region.

### 4.4 Y‐Maze Task

The Y‐maze assesses spatial working memory, interpreted by the percent of spontaneous alternations made during the testing period. The maze was made of black‐painted wood and each arm was 35 cm long, 14 cm high, 5 cm wide, and positioned at equal angles. Mice were randomly placed at the end of one of the 3 arms to avoid placement bias and allowed to freely explore the Y‐maze during an 8 min session. The series of arm entries was recorded visually and arm entry was considered to be completed when the hind paws of the mouse were completely placed in the arm. Alternation was defined as successive entries into the three arms on overlapping triplet sets. The percentage alternation was calculated as the ratio of actual to possible alternations (defined as the total number of arm entries minus 2), multiplied by 100.

### 4.5 Morris Water Maze

The MWM was performed mostly as described [44]. During the training session, a transparent rescue platform was submerged under the painted water (0.5–1 cm) and was placed in a fixed position in the pool. On the first day of training, mice were first allowed to stand on the platform for 10 s. After that, mice were gently placed into the water facing the wall of the pool and allowed to freely explore the whole maze for 60s. Mice were then guided to the rescue platform if they did not find it. Mice were allowed to take a rest on the platform for 10s and then retrained from a different start position with the same procedure. The body temperature of the mice was maintained at 37°C using animal warmers after every trial. 24 h later, mice were trained again following the same procedure without the initial habituation session. Mice were trained for 5 consecutive days. On day 6, mice were put into the water maze for 1 min, where the platform had been removed. Mouse behaviors were videotaped and analyzed by the Noldus EthoVision XT 13.0. The MWM was virtually divided into four quadrants. The rescue platform was in the target quadrant. Escape latency was defined as the time spent before finding the platform. Escape latency during the 5‐day training sessions served as an independent measurement of spatial learning and memory.

### 4.6 Step‐Down Passive Avoidance Test

The step‐down passive avoidance (SDA) test was performed to assess long‐term aversive memory. Briefly, in the training trial, animals were placed on the platform and their latency to step down on the grid with all four paws was measured. Immediately after the stepping down on the grid, the animals received a single mild foot shock (0.4 mA, 2.0 s). A retention test trial was performed 24 h after the training section. The results were expressed as latency period to step down the platform, with a cut‐off at 300 s.

### 4.7 Circadian Activity Measurements

Mice were placed in cages with a 4.72‐inch running wheel, and their activity was monitored in 1‐min bins using the Running Counter system (RWD Life Science, Shenzhen, China), and cages were changed every 2 weeks. Free‐running circadian period (tau) was calculated with ActogramJ [62]. NC‐F1 and LL‐F1 male mice were placed under 12h‐light/12h‐dark cycles for 7 days before placed in constant darkness (DD) without any exposure to light. Activity was recorded in constant darkness for 15 days.

### 4.8 Novel Object Recognition Test

Novel object recognition (NOR) test was carried out in a specified open field box (42 × 42 × 30 cm). The test involves first habituation of mice to explore in empty open field box followed by training session in the same open field box with novel objects. Habituation was carried out for 2 sessions (8 min each) per day for 3 successive days. Mice were then allowed to explore two identical novel objects that were placed into the open field box 14 cm away from each other. Each mouse was given 5 min to explore both objects and then returned back to their home cage. 24 h later, one of the similar objects was replaced by a novel object with different color and shape, and mice were permitted to explore them for 6 min. Time and frequency spent on familiar and novel object was recorded. Animal behavior during the training and test session was tracked by a top camera and analyzed by Noldus software. Discrimination index (DI) was calculated by the following formula: (Time exploring novel object – Time exploring familiar object) / Total object exploration time. Higher DI indicates better recognition memory.

### 4.9 Elevated Plus Maze

The elevated plus maze (EPM) test was performed to assess anxiety like behavior. Mice were placed at the center of a plus shaped apparatus elevated 50–60 cm above the floor, containing two open arms and two closed arms of equal dimensions. Each mouse was allowed to explore freely for 5 min, and behavior was recorded using an automated video tracking system. The primary outcome was the percentage of time spent in the open arms, calculated as 100 × (open‐arm time / (open‐arm time + closed‐arm time)).

### 4.10 Electrophysiology

Mice were anesthetized by ketamine/xylazine (Sigma) and perfused transcardially for 1 min with 4°C modified artificial cerebrospinal fluid (ACSF) containing (in mm) 250 glycerol, 2 KCl, 10 MgSO_4_, 0.2 CaCl_2_, 1.3 NaH2PO_4_, 26 NaHCO_3_, and 10 glucose, to protect CNS neurons and maintain functional connectivity of brain slices. Mice were then decapitated, and brains were quickly removed and chilled in ice‐cold ACSF for additional 1 min. Transverse hippocampal slices (350 µm) were prepared using a Vibroslice (VT 1000S; Leica) in ice‐cold ACSF. Slices were then incubated in regular ASCF containing (in mm): 126 NaCl, 3 KCl, 1.25 NaH_2_PO_4_, 1.0 MgSO_4_, 2.0 CaCl_2_, 26 NaHCO_3_, and 10 Glucose for 30 min at 34°C for recovery, and then at room temperature (25 ± 1°C) for an additional 2–8 h. All solutions were saturated with 95% O2/5% CO2 (vol/vol). Dox (10 ng/ml) was present in the perfusate for experiments with slices from aDox‐treated mice. Slices were placed in the recording chamber that was perfused (3 mL/min) with ACSF at 32°C–34°C. Whole‐cell patch‐clamp recordings from CA1 neurons were aided with infrared optics using an upright microscope equipped with a ×40 water‐immersion lens (Olympus, BX51WI) and infrared‐sensitive CCD camera. And all data were recorded in voltage‐clamp configuration by the HEKAEPC10 amplifier.

For recording mEPSCs, we block GABAA receptors with 20 µm bicuculline methodiod (BMI) and action potentials with 1 µm TTX in ACSF, respectively. The pipettes (input resistance: 4–6 MΩ) solution contained (mm): 105 K‐gluconate, 30 KCl, 10 HEPES, 10 phosphocreatine, 4 ATP‐Mg, 0.3 GTP‐Na, 0.3 EGTA, 5 QX314 (pH 7.35, 285 mOsm). EPSCs were evoked by electrical stimulation of axons in the stratum radiatum (0.05 Hz) in the presence of BMI (20 µm), and were verified by adding DL‐AP5 (100 µm) and CNQX (20 µm). The pipette (input resistance: 2–4 MΩ) solution contained (in mm) 135 Cs‐methanesulfonate (CsCH_3_SO_3_), 8 NaCl, 10 Hepes, 10 phosphocreatine, 4 ATP‐Mg, 0.3 GTP‐Na, 0.3 EGTA, and 5 mm QX314 (pH, 7.3, 290 mOsm).

For recording mIPSCs at the holding potential of ‐70 mV, the concentration of CsCl was increased to 140 mm, and CsCH_3_SO_3_ was omitted to enhance the driving force of Cl–, and 1 µm TTX was added in the bath solution. Data were collected when series resistance fluctuated within 20% of initial values, filtered at 1 kHz, and sampled at 10 kHz.

For recording long‐term potentiation (LTP), coronal hippocampal slices (300 µm) were cut in ice‐cold modified ACSF with a VT‐1000S vibratome (Leica, Germany) and transferred to an incubation chamber containing regular ACSF (in mM) (126NaCl, 3 KCl, 1 MgSO_4_, 2 CaCl_2_, 1.25 NaH_2_PO_4_, 26 NaHCO_3_, and 10 glucose) at 32°C for 30 min and at room temperature (25 ± 1°C) for additional 1 h before recording. All solutions were saturated with 95% O_2_/5% CO_2_ (vol/vol). Slices were placed in the recording chamber, which was superfused continuously with ACSF (2 mL/min). Slices were visualized with infrared optics using an upright microscope equipped with an infrared‐sensitive CCD camera (DAGE‐MTI, IR‐1000E). The pipettes were pulled by a micropipette puller (P‐97, Sutter instrument) with a resistance of 3–5 MΩ. Recordings were made with a MultiClamp700B amplifier and 1,440A digitizer (Molecular Device), data were filtered at 1 kHz and sampled at 10 kHz. The Schaffer Collaterals (SC)‐CA1 pathway was stimulated with a concentric bipolar electrode (FHC), and field excitatory postsynaptic potentials (fEPSPs) were recorded in current‐clamp with ACSF‐filled glass pipettes (1–5 MΩ). Monophasic 100‐µs pulses of constant currents with intensity was used as stimuli and adjusted to produce∼30% of maximal amplitudes, at a frequency of 0.033 Hz. The strength of synaptic transmission was determined by measuring the initial (10%‐60% rising phase) slope of fEPSPs. LTP was induced by two trains of 100 pulses in 1 sec, at an interval of 20 s. The level of LTP was determined at an average of 50–60 min after tetanus stimulation.

### 4.11 LC‐MS/MS Analysis

The quantification of hippocampi neurotransmitters, including histamine, GABA, DOPA, 5‐hydroxyindoleacetic acid (5‐HIAA), norepinephrine (NE), glutamate (Glu), glutamine (Gln), serotonin, tyramine, norepinephrine, normetanephrine, and acetylcholine was performed using liquid chromatography‐tandem mass spectrometry (LC‐MS/MS). Analysis was run on an Agilent 6410 triple stage quadrupole mass spectrometer equipped with an ESI ion source and an Agilent 1290 HPLC system with an auto‐sampler (Agilent Technologies, Santa Clara, CA, USA). Briefly, the analytes were separated on a BEH C18 column (2.1 mm × 100 mm, 1.7 µm, Waters, Milford, USA) at 30°C. The mobile phase, consisting of 0.1% formic acid in water (Solvent A) and acetonitrile (Solvent B), was used with a gradient elution of 0–4 min, 2% B; 6 min, 80% B; 8–10 min, 90% B at a flowrate of 0.1 mL/min. ESI‐MS/MS conditions were set as follows: gas temperature 325°C, gas flow 10 min, capillary 4000 V, nebulizer pressure 35 psi. MS acquisition of neurotransmitters was performed in electrospray positive ionization multiple reaction monitoring (MRM) mode.

### 4.12 Bulk RNA‐seq and Data Analysis

Total RNA was extracted using TRIzol (Invitrogen) followed by DNase I treatment. RNA quantity was measured using Nanodrop 2000 (Thermo Fisher), and RNA integrity was assessed using the Agilent 2100 Bioanalyzer with the RNA Nano 6000 assay (Agilent Technologies). Only samples with RNA integrity number (RIN) ≥ 7.0 were used for library construction.

For bulk RNA‐seq, polyadenylated mRNA was enriched using oligo‐dT magnetic beads and fragmented under high temperature in First‐Strand Synthesis Reaction Buffer. First‐strand cDNA was synthesized using random hexamer primers and M‐MuLV reverse transcriptase, followed by second‐strand synthesis with DNA Polymerase I and RNase H. Double‐stranded cDNA underwent end‐repair, A‐tailing, and ligation of Illumina sequencing adapters with hairpin‐loop structures. Size selection (∼370–420 bp) was performed using AMPure XP beads (Beckman Coulter). Libraries were amplified using Phusion High‐Fidelity DNA polymerase, purified, and quality‐checked using the Agilent 2100 Bioanalyzer. Cluster generation was performed on a cBot instrument using a TruSeq PE Cluster Kit (Illumina). Paired‐end sequencing (150 bp × 2) was carried out on an Illumina NovaSeq platform.

Read quality was assessed using FastQC and all the downstream analyses were based on the clean data with high quality. Clean reads were mapped to Mus musculus genome (mm10) using Hisat2v2.0. feature Counts v1.5.0‐p3 was used to count the reads numbers mapped to each gene. And then FPKM of each gene was calculated based on the length of the gene and reads count mapped to this gene. Normalization and differential expression (DE) analysis were performed with DESeq2. The resulting *p*‐values were adjusted using the Benjamini–Hochberg method to control the false discovery rate. For broad pattern identification and downstream candidate selection, genes with a nominal *p*‐value < 0.05 and |log2FC| > 1 were retained for further analysis.

ClusterProfiler v3.14.3 was used for the functional description of the DEGs and Gene Set enrichment Analysis (GSEA). A preranked analysis was performed using log2FC as a ranking metric in GSEA. Only gene sets with a |NES|> 1 and *p‐*value < 0.05 were considered for descriptive analysis.

### 4.13 Western Blot

Proteins of hippocampi were extracted using RIPA Lysis Buffer (P0013B, Beyotime Biotechnology, China) with 1% Protease Inhibitor Cocktail (20124ES03, Yeasen, China) and 1% Phosphate Inhibitor Cocktail (20109ES05, Yeasen, China), homogenized 30 s in 70 Hz twice. Proteins were separated with SDS–PAGE and then transferred to PVDF membranes. After blocking with 5% non‐fat milk, membranes were incubated with primary antibodies at 4°C overnight and secondary antibodies (1:3000, 7076 and 7074, CST, USA) at room temperature for 1 h. Western blots were imaged on a ChemiDoc Imaging system (Bio‐Rad) and band intensities quantified using ImageJ. Uncropped Western blot images are provided in Data S10.

### 4.14 Immunofluorescence Staining

Anesthetized mice were perfused transcardially with PBS and 4% PFA. Tissues were fixed in 4% PFA at 4°C for 8 h. After dehydration by 30% sucrose, brain blocks were frozen and cut into 30‐µm‐thickcoronal sections on the Leica CM1950 cryostat slicer. Sections were permeabilized with 0.2% Triton X‐100 and 3% BSA in PBS and incubated with primary antibodies at 4°C overnight. After washing with PBS for 3 times, samples were incubated with Alexa Fluor‐conjugated secondary antibodies (1:800, Abcam) for 1 h at room temperature. Nuclei were stained using DAPI. Samples were mounted with Vectashield mounting medium (H‐1200, Vector Lab) and images were taken by Leica SP8 confocal microscope. Images were analyzed using ImageJ software.

### 4.15 Corticosterone Assay

Mice serum was sampled by extracting the eyeball blood after mice were anesthetized with isoflurane. Blood was collected into tubes and left to clot (30 min, room temperature). Then the samples were centrifuged to isolate the serum (10 min, 4°C, 2000 × g) and kept at −80°C until assay. Blood collection was performed every 4 h across a 24‐h cycle at 08:00, 12:00, 16:00, 20:00, 00:00, and 04:00. The concentration of corticosterone was determined using an ELISA kit (#K014 – H1; Arbor Assays, USA) following the manufacturer's instructions.

### 4.16 Energy Expenditure

Energy expenditure and metabolic parameters were assessed using a Comprehensive Lab Animal Monitoring System (CLAMS; Columbus Instruments). Mice were individually housed in metabolic chambers with free access to food and water for 72 h, including an initial 12 h acclimation period. Oxygen consumption (VO_2_; mL/kg·h) and respiratory exchange ratio (RER = VCO_2_/VO_2_) were continuously recorded. Activity was measured as XTOT counts. Food intake was monitored by the automated feeding sensor integrated in the system and quantified as cumulative intake per mouse over time. All parameters were normalized to body weight where appropriate. Data were extracted at 1‐hour intervals to generate time series plot across light and dark phases.

### 4.17 Mouse Sperm Sample Collection

Mature sperm were extracted from the cauda epididymis of 14‐week‐old F0 male mice. The cauda epididymis was dissected from mice and placed in BWW media to collect mature sperm. Three small incisions were made at the cauda and epididymis were gently squeezed to allow the caudal fluid to ooze out. Sperm‐containing media was incubated for 15 min at 37°C, then transferred to a fresh tube. After incubation sperm were collected by centrifugation at 700 g for 3 min, followed by a 1X PBS wash, and a second wash with somatic lysis buffer (0.1% SDS, 0.5% Triton X‐100 in DEPC H_2_O) for 20 min on ice to eliminate somatic cell contamination. Somatic lysis buffer treated sperm were collected by centrifugation at 800 g for 5 min, and finally washed with 1X PBS twice before storage at −80°C. Microscopic evaluation confirmed >99% sperm purity, with complete absence of somatic cells.

### 4.18 Epididymal Fluid Collection

Bilateral caudal epididymides from 14‐week‐old F0 mice were isolated and minced with fine scissors in 450 uL HTF medium. The tissue suspension was then incubated in 1.5‐mL centrifuge tube for 15 min at 37°C to allow the diffusion of luminal content. Subsequently, the sample was subjected to high‐speed centrifugation (10 000 × g for 10 min) to pellet tissue debris and sperm. The clear supernatant was collected, defined as the epididymal fluid, and stored at −80°C until further use.

### 4.19 Isolation of Spermatogonial Stem Cells

Testes were collected in ice‐cold PBS, and the tunica albuginea was removed. Seminiferous tubules were incubated in 5 mL DMEM containing collagenase type IV (1 mg/mL) and DNase I (150 µg/mL) at 37°C for 30 min with gentle agitation. The dispersed tubules were further digested with 1× TrypLE (Gibco, A1217701) at 37°C for 11 min with gentle pipetting, and the reaction was terminated by adding 0.5 mL fetal bovine serum (FBS). The cell suspension was passed through a 40‐µm mesh filter and centrifuged at 160 g for 10 min at 4°C.

Cells were resuspended at 1 × 10^6^ cells/mL in DMEM containing Anti‐Biotin MicroBeads (Miltenyi Biotec, 130‐090‐485) and anti‐mouse CD117 antibody (1:200, eBioscience 13–1171) and incubated at 37°C for 30 min. Magnetic separation was performed using an autoMACS Pro system, and the CD117^−^ fraction was collected. For FACS enrichment, cells were incubated with anti‐mouse CD9 (1:100, eBioscience 17–0091) and anti‐mouse CD90.2 (1:200, eBioscience 11–0902) antibodies at 37°C for 10 min, followed by sorting on a BD FACSMelody. Dead cells were excluded by DAPI staining. SSCs were isolated as CD9^+^CD90.2^+^ populations.

The isolation strategy used in this study is consistent with previously reported SSCs purification methods [63]. Immediately after sorting, SSCs were processed in parallel for bulk RNA‐seq, ATAC‐seq, and small RNA‐seq library preparation to preserve RNA integrity and chromatin accessibility. No freezing or intermediate storage steps were applied. SSCs purity was confirmed based on FACS gating profiles and marker specificity.

### 4.20 Isolation of Inner Cell Mass

Our ICM isolation method was based on microsurgery with an inverted microscope (Olympus IX71) equipped with micromanipulators (Narishige ON4). After removal of the zona pellucida by incubation in pre‐warmed 0.5% Pronase E solution (Sigma, P8811) on a 37°C for 5 min, blastocysts were placed in 0.05% Trypsin (Gibco, 25300054) by pipetting gently several times at 37°C for 5 min and then terminated by adding fetal bovine serum (FBS) to inactivate Trypsin. The dissociated cell was collected by micromanipulators based on their morphology. Each ICM was collected as an individual sample and immediately processed for Smart‐seq2 library preparation.

### 4.21 Luciferase Reporter Assay

To validate direct targeting by miR‐92a‐3p and miR‐25‐3p, the predicted seed binding regions of the *Cyp51* and *Dlg4* 3′UTRs were cloned downstream of the Firefly luciferase gene in the pmirGLO vector (GeneChem, Shanghai, China). Corresponding mutant (Mut) reporters were generated by disrupting the seed‐match sequences. HEK293 cells were cotransfected with the WT or Mut pmirGLO reporters together with miR‐92a‐3p or miR‐25‐3p expression plasmids (empty vector as control) using Lipofectamine. After 48 h, Firefly and Renilla luciferase activities were measured using the Dual‐Luciferase Reporter Assay System Kits (Promega). Relative luciferase activity (Firefly/Renilla) was normalized to control samples.

### 4.22 ATAC‐seq and Data Analysis

ATAC‐seq was performed on purified spermatogonial stem cells (SSCs) isolated from control and constant‐light–exposed male mice. Approximately 2–4 × 10^4^ SSCs per sample were collected, washed with PBS, and processed using a transposase‐based ATAC‐seq library preparation kit according to the manufacturer's low‐input protocol. Briefly, SSCs were lysed to release nuclei, followed by Tn5‐mediated tagmentation. Tagmented DNA fragments were purified and PCR‐amplified to generate sequencing libraries. Libraries were sequenced on an Illumina NovaSeq 6000 platform. Two biological replicates were analyzed for each condition.

Raw FASTQ files were quality‐trimmed using Trim Galore to remove adapters and low‐quality bases. Processed reads were aligned to the mm10 mouse reference genome using Bowtie2 with default parameters. PCR duplicates were removed using Picard MarkDuplicates, and mitochondrial reads were filtered out. To account for the offset introduced by Tn5 tagmentation, aligned reads were adjusted by shifting +4 bp on the positive strand and −5 bp on the negative strand. Peak calling was performed using MACS2 with parameters optimized for ATAC‐seq. Normalized chromatin accessibility tracks (bigWig files) were generated using bamCoverage (–binSize 10 –normalizeUsing RPKM –extendReads) and visualized in Integrative Genomics Viewer (IGV). Because the experiment included *n* = 2 biological replicates per group, ATAC‐seq results were used to qualitatively assess changes in chromatin accessibility rather than to perform statistical identification of differentially accessible regions (DARs).

### 4.23 Smart‐seq2 and Data Analysis

Total RNA was extracted from single embryos at the 1‐cell (1C), 2‐cell (2C), and 8‐cell (8C) stages, as well as from individually isolated inner cell masses (ICMs), using a low‐input RNA extraction protocol RNA Extraction CZ Kit (RNC643, ONREW) according to the manufacturer's instructions. RNA quantity was assessed using a Qubit 4.0 fluorometer (Invitrogen), and RNA integrity for low‐input samples was verified by electrophoresis.

Full‐length cDNA synthesis and preamplification were performed using the Smart‐seq2–based Single Cell Full‐Length mRNA Amplification Kit (Vazyme, N712‐03), following the optimized protocol for single‐embryo and low‐input samples. Amplified cDNA was purified with VAHTS DNA Clean Beads (Vazyme), quantified, and subsequently used to construct sequencing libraries through a tagmentation‐based low‐input DNA library preparation workflow. Libraries were sequenced on an Illumina NovaSeq 6000 platform with paired‐end 150 bp reads.

Sequencing quality was assessed using FastQC, and adapters or low‐quality reads were removed using Trim Galore. Filtered reads were mapped to the mm10 mouse reference genome using STAR in two pass mode, and only uniquely mapped reads were retained. Gene‐level quantification was performed using featureCounts, and differential expression analysis across developmental stages or treatment groups was carried out using DESeq2. For broad pattern identification and downstream candidate selection, genes with a nominal *p*‐value < 0.05 and |log2FC| > 1 were retained for further analysis. Gene Ontology (GO) enrichment of differentially expressed genes was conducted using clusterProfiler with the org.Mm.eg.db annotation package, and biological processes with *p*‐value < 0.05 were considered significantly enriched.

### 4.24 Human Sample Inclusion Criteria and Collection

This study was approved by the Ethics Committee of the International Peace Maternity and Child Health Hospital, Shanghai Jiao Tong University School of Medicine (approval number: (GKLW)2018‐01) and Shanghai First Maternity and Infant Hospital (approval number: KS25461). Informed consent was obtained for all the procedures. Human semen samples were obtained from reproductive‐age Chinese men under standardized morning collection procedures after 2–7 days of abstinence before any clinical treatment. An initial pilot cohort (10 NC vs. 10 CD) was generated, but due to inter‐individual variability and the inability to standardize shift‐work schedules, a larger validation cohort was established. In the final cohort (25 NC vs. 25 CD), individuals with night‐shift work or recent cross‐time zone travelers were excluded. All participants completed a sleep questionnaire covering weekday and weekend sleep timing, day‐to‐day regularity. Circadian disrupted (human‐CD) participants were defined as having a late chronotype, characterized by average weekday sleep onset after 00:00, together with at least one additional circadian misalignment feature, including social jetlag greater than 2 h (difference between weekend and weekday sleep onset times) or highly irregular sleep timing, reflected by typical day‐to‐day variations in sleep onset timing exceeding 90 min. Control (human‐NC) participants exhibited stable sleep–wake schedules with average sleep onset before 23:00 and social jetlag less than 1 h.

For all participants, inclusion criteria were age 20–45 years, abstinence duration 2–7 days, and normal semen parameters according to WHO 2010 guidelines. Exclusion criteria included chronic illness, endocrine disorders, recent infection, and use of medications known to affect spermatogenesis. Detailed participant characteristics are provided in Table S3.

Upon collection, samples were allowed to liquefy for 30 min at 37°C and were processed immediately thereafter. Liquefied semen was centrifuged at 3000 rpm for 5 min to remove the seminal plasma. The sperm pellet was resuspended in sterile PBS (0.5 M) and washed twice under the same centrifugation conditions to eliminate residual contaminants. After the final wash, sperm pellets were snap‐frozen and stored at −80°C until downstream analyses, including RNA extraction and small RNA quantification.

### 4.25 Small RNA Library Preparation and Sequencing

Preparing cDNA library of small RNAs for deep sequencing was performed as previously described [64]. Total RNA from purified mouse sperm, spermatogonial stem cells (SSCs), or human spermatozoa was extracted using TRIzol reagent under RNase‐free conditions. The quantity of isolated RNA was measured with Qubit 2.0 Fluorometer (Invitrogen). Small RNA libraries were generated using an improved low‐input protocol optimized for quantitative recovery of miRNAs, tsRNAs, rsRNAs, and other 18–40 nt species. For library construction, approximately 50 ng (human) or 100 ng (mouse) of total RNA was used. Briefly, 3′ adapters were ligated to small RNAs using a pre‐adenylated oligonucleotide and truncated RNA ligase, followed by 5′ adapter ligation, first‐strand cDNA synthesis, and limited cycle PCR amplification. Adapter ligated cDNA fragments corresponding to 18–40 nt small RNAs were size‐selected by PAGE purification to remove adapter dimers and nonspecific products. Libraries passing quality control were sequenced on an Illumina NovaSeq platform (PE150) for human samples and on the HiSeq X Ten platform for mouse samples.

### 4.26 Analysis of Small RNA Expression

Raw sequencing reads underwent adapter trimming and quality filtering with cutadapt. Reads ≥17 nt after trimming were retained for downstream analysis. Clean reads were aligned to reference databases of miRNAs, tRNAs, rRNAs, and other small RNA species with stringent parameters to ensure high‐confidence annotation. miRNA quantification was performed by counting reads mapped to annotated mature miRNA loci (miRBase v21), allowing a ± 2‐nt extension at the 3′ end when templated by the genome. Expression levels were normalized as reads per million mapped reads (RPM). Differential expression analysis was conducted using DESeq2. Adjusted *p*‐values < 0.05 were used to define statistical significance, whereas nominal *p*‐value < 0.05 was applied as a screening threshold for broad pattern identification.

### 4.27 miRNA‐mRNA Target Predictions

MultimiR (v.1.8.0) was used to find validated and predicted miRNA‐gene target. We only considered miRNA–target pairs that were either functionally validated in at least one resource or that were predicted in at least two databases out of the 11 databases that we queried (miRecords, miRTarBase, TarBase, DIANA‐microT‐CDS, ElMMo, MicroCosm, miRanda, miRDB, PicTar, PITA and TargetScan).

### 4.28 RNA Isolation and Quantitative RT‐PCR of mRNA and miRNA

Total RNA from tissues and sperm was extracted using TRIzol Reagent (Invitrogen) following the manufacturer's instructions. For mRNA quantification, 1 µg of total RNA was reverse transcribed using reverse transcriptase (Vazym, R323). Quantitative PCR was performed using a SYBR Green PCR kit on a QuantStudio 5 Real‐Time PCR System (Life Technologies). Relative mRNA expression levels were calculated using the ΔΔCt method and normalized to Rplp0/Gapdh/18S, which served as internal reference genes. Primer sequences are provided in Data S11.

For miRNA quantification, 500 ng of total RNA was reverse transcribed using reverse transcriptase (Accurate Biotechnology, AG11743) and specific stem‐loop RT primers (Sangon). Quantitative PCR was carried out using the SYBR Green PCR kit on the QuantStudio 5 system. miRNA expression levels were normalized to U6 snRNA using the ΔΔCt method.

### 4.29 Zygote Injection, Culture, Embryo Transfer, and Collection

Total sperm RNA was extracted using the mirVana miRNA Isolation Kit (AM1561, Thermo Fisher Scientific). And small RNA (<200 nt) fractions were further separated according to the manufacturer's protocol, with strict RNase‐free handling throughout the procedure. The integrity and size distribution of small RNA were verified by PAGE prior to microinjection. Total RNA, small RNA, or microRNA mimics were prepared at the concentrations required for each experiment. For offspring‐generation experiments, microRNA mimics were used at 2 ng/µL. C57BL/6 female mice were induced ovulation and mated with the male mice. One‐cell‐stage embryos were collected for injection. Each group was micro‐injected into embryos via using Eppendorf FemtoJet microinjection system. The injected embryos were cultured in KSOM (Sigma–Aldrich, MR‐106‐D) at 37°C in 5% CO_2_ conditions. Approximately 300 embryos were injected for each condition, and 90% implanted embryos survived up to two‐cell stage. The two‐cell embryos were transferred into the oviducts of the surrogate mothers of the ICR background (twelve embryos for each side oviduct).

For transcriptional profiling, embryos at the 1‐cell (1C), 2‐cell (2C), and 8‐cell (8C) stages, as well as inner cell masses (ICMs), were collected at their corresponding developmental time points. Immediately after collection, individual embryos or ICMs were placed into lysis buffer and processed directly for Smart‐seq2 full‐length cDNA synthesis, without freezing or intermediate storage, following the Single Cell Full‐Length mRNA Amplification Kit protocol (Vazyme, N712‐03).

### 4.30 Embryo Immunofluorescence and Dose‐Titration Analysis

For dose‐titration experiments, zygotes were microinjected with scramble control mimics or synthetic miR‐92a‐3p/miR‐25‐3p mimics at 0.2, 1, or 2 ng/µL, corresponding approximately to 1‐, 5‐, and 10‐sperm equivalents, respectively. Injected embryos were cultured in KSOM at 37°C under 5% CO_2_ until the early morula stage. Embryos were then fixed in 4% paraformaldehyde, permeabilized, blocked, and incubated with anti‐CYP51 primary antibody followed by the fluorescent secondary antibody. Nuclei were counterstained with DAPI. Images were acquired under identical confocal settings for all groups. CYP51 fluorescence intensity was quantified in ImageJ as mean pixel intensity within the embryo region after background subtraction and normalized to the scramble control group.

### 4.31 Reagents and Antibodies

Chemicals were purchased from Sigma‐Aldrich unless otherwise indicated. Information of antibodies was as follows: rabbit anti‐BDNF (Abcam) (ab108319; 1:1,000 for blotting); rabbit anti‐PSD95 (CST)(2507; 1:1000 for blotting); rabbit anti‐GABAAα1(Millipore)(06‐868; 1:2000 for blotting); rabbit anti‐ GABAAβ1/2/3 (Santa Cruz)(sc‐28794; 1:1000 for blotting); rabbit anti‐GluR1 (Abcam)(ab109450; 1:2000 for blotting); mouse anti‐GluR2 (Millipore)(MABN71; 1:2000 for blotting); rabbit anti‐NR2A (Millipore)(05‐901R; 1:2000 for blotting); rabbit anti‐NR2B (Millipore)(ab65783; 1:2000 for blotting); HRP‐conjugated β‐Actin (ABclonal)(AC028; 1:200000 for blotting); rabbit anti‐Synapsin I (Abcam) (ab254349; 1:500 for staining); Guinea pig anti‐PSD95 (oasisbiofarm) (OB‐PGP053; 1:500 for staining); rabbit anti‐CYP51 (Proteintech)(13431‐1‐AP; 1:500 for staining).

### 4.32 Statistics

Data are shown as the mean ± SEM. Statistical significance was defined as specified for each analysis in the corresponding Methods and figure legends. For sequencing‐based analyses, nominal *p*‐value and fold‐change cutoffs were used for candidate selection, whereas adjusted *p*‐value was used where indicated to assess statistical significance. Behavioral data were analyzed using mixed‐effects models treating litter as a random factor, with repeated water maze training modeled using Group × Day interactions. GO analysis was performed using a hypergeometric distribution test. The statistical test utilized by GSEA is the Kolmogorov‐Smirnov statistical test. All the statistical analyses were conducted with GraphPad Prism 10 (GraphPad Software, Inc) and R (version 4.3.1).

## Author Contributions

G.D. designed the research. K.Z., Y.D., S.L., B.T., Z.L., Y.Z., Y.M., Z.W., W.S., C.Y., Z.L., S.W., M.Z., Y.L., J.Y., J.M., C.Z. and X.Q. performed the experiments. G.D., Y.D., K.Z. analyzed and interpreted the data. H.H., J.L., Y.Z., and J.S. provided platform and technical support. G.D., Y.D., Z.S., and K.Z. wrote the draft of the manuscript. All authors read, amended the manuscript, and approved its final version.

## Conflicts of Interest

The authors declare no conflicts of interest.

## Supporting information




**Supporting File 1**: advs75462‐sup‐0001‐SuppMat.docx.

**Supporting File 2**: advs75462‐sup‐0002‐data.zip.

## Data Availability

There are no restrictions on data availability. The raw sequencing data was deposited in Gene Expression Omnibus (GEO). All data generated or analyzed during this study are included in this published article and its supplementary files or are available upon request.
