# Supplementary material for: Paternal Circadian Disruption Impairs Offspring Cognition via Sperm microRNAs
Source: Adv Sci (Weinh). 2026 Apr 28:e14510. Online ahead of print. doi: 10.1002/advs.202514510 (PMC13334623; doi:10.1002/advs.202514510)
Supplement: Supplementary file 1 — Supporting File 1: advs75462‐sup‐0001‐SuppMat.docx. [file ADVS-9999-e14510-s002.docx]

Supporting Information

Title: Paternal Circadian Disruption Impairs Offspring Cognition via Sperm microRNAs

*Kexin Zou*, *Sisi Luo*, *Binliang Tang*, *Zhiqiang Liu*, *Yuchuan Zhou*, *Yicong Meng*, *Zhengmu Wu*, *Zheng Sun*, *Weihui Shi*, *Jianzhong Sheng*, *Chuanjin Yu*, *Jinsong Li*, *Hefeng Huang*, *Yifu Ding**, *Guolian Ding**

**Table S1: Mouse sperm parameter assessed by CASA.**

|  | NC-F0 | LL-F0 |
| --- | --- | --- |
| Concentration (M/ml) | 36.9±3.2 | 38.5±3.8 |
| Motile (%) | 86.6±2.4 | 85.3±0.8 |
| Progressive (%) | 31.4±1.1 | 31.5±0.6 |
| VAP (μm/s) | 81.9±2.4 | 76.3±2.6 |
| VSL (μm/s) | 66.6±1.9 | 63.3±2.0 |
| VCL (μm/s) | 121.6±3.8 | 114.4±4.4 |
| ALH (μm) | 5.9±0.2 | 5.7±0.2 |
| BCF (Hz) | 13.0±1.2 | 14.7±0.5 |
| STR (%) | 80.8±0.5 | 83.0±0.6 |
| LIN (%) | 57.2±1.1 | 58.3±0.9 |
| Elongation (%) | 39.0±0.4 | 40.5±0.6 |
| Area (μm sq) | 6.0±0.2 | 5.8±0.1 |

The sperm motility, viability and corresponding parameters were analyzed by computer-aided sperm analysis (CASA) *In vitro*.

Data were presented as mean±SEM. VAP, Path Velocity; VSL, Prog. Velocity; VCL, Track Speed; ALH, Lateral Amplitude; BCF, Beat Frequency; STR, Straightness; LIN, Linearity. n _NC-F0_=5, n _LL-F0_=4.

**Table S2: LL mice display fertility parameters similar to those of control mice.**

|  | NC-F0 | LL-F0 |
| --- | --- | --- |
| Plug-positive females (%) | 78.6(22/28) | 80.0(24/30) |
| Pregnancy rate (%) | 57.1(16/28) | 56.7(17/30) |
| litter size (n) | 6.6±0.53 | 6.3±0.52 |

Male mice of last light (LL-F0) and normal control (NC-F0) were mated with C57BL/6J adult female WT mice. The pregnancy rate and natural born offspring were counted. Data were presented as mean±SEM.

**Table S3** Characteristics of human participants in the NC and CD groups

|  | human-NC  (n = 25) | human-CD  (n = 25) | *P* value |
| --- | --- | --- | --- |
| Age (years) | 31.8±0.7 | 31.6±0.7 | 0.76 |
| BMI (kg/m2) | 25.0±0.4 | 24.2±0.5 | 0.31 |
| Forward motility rate (%) | 37.9±3.1 | 34.2±2.2 | 0.53 |
| Sperm concentration (million/mL) | 40.3±6.2 | 32.2±4.0 | 0.33 |

Data are presented as mean ± SEM. Normality was assessed using the Shapiro–Wilk test. Variables with normal distribution were compared using unpaired two-tailed t-tests. Non-normally distributed variables were analyzed using Mann–Whitney U tests. Semen parameters were obtained from the hospital clinical laboratory, where routine semen analysis was performed by certified andrology technicians following WHO (2010) guidelines.


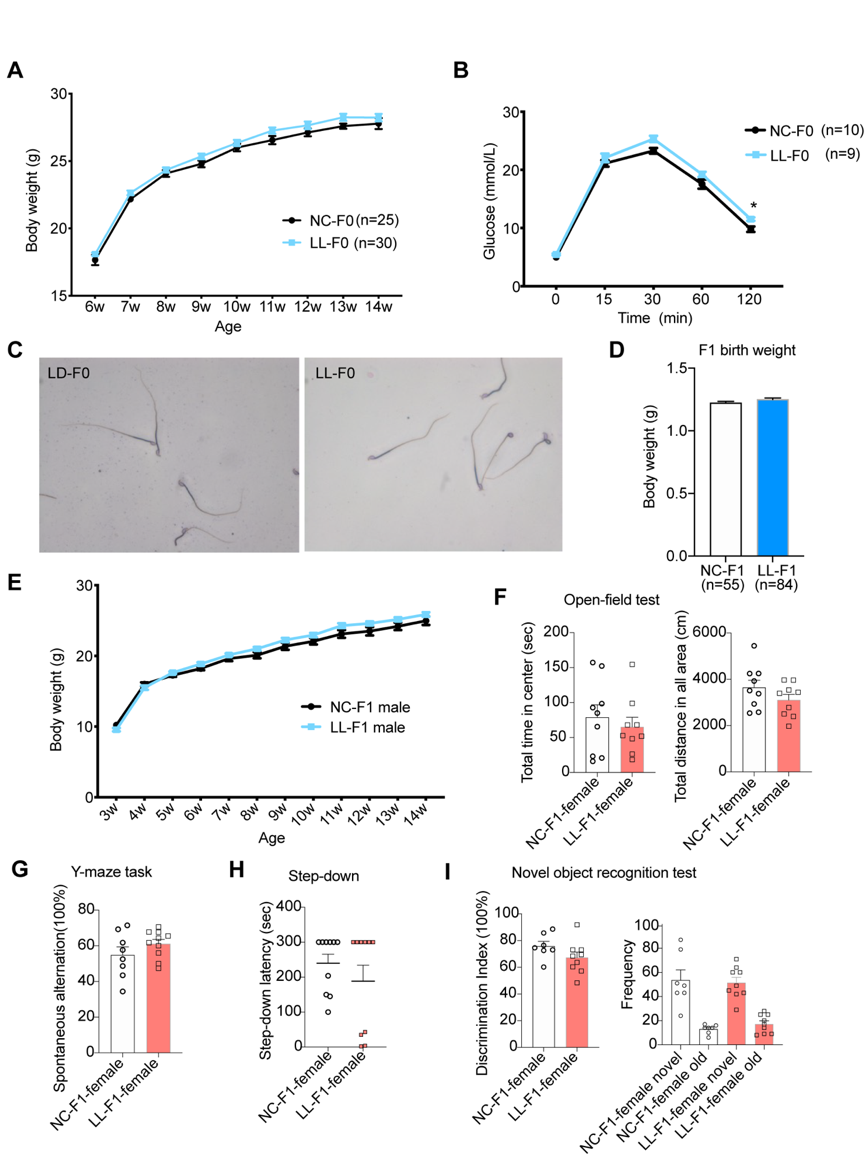


**Figure S1. Physiological and behavioral assessments in F0 males and F1 offspring.**

(A) Mean body weight over time of LL-F0 and NC-F0. n_NC-F0_ = 25, n_LL-F0_ = 30 mice.

(B) GTT of mice exposed to constant light (LL-F0) or 12h-light/12h-dark cycles (NC-F0). n_NC-F0_ = 10, n_LL-F0_ = 9 mice.

(C) Giemsa staining of F0 mouse sperm. Giemsa staining of spermatozoa from F0 males (representative images; n = 3 per group).

(D) Birth weight of F1 male offspring. n_NC-F1-male_ = 55, n_LL-F1-male_ = 84 mice.

(E) Body weight of F1 male offspring from 3 to 14 weeks after birth. n_NC-F1-male_ = 17-31, n_LL-F1-male_ = 9-22 mice.

(F-I) Behavioral test of F1 female offspring. n_NC-F1-female_ = 7-10, n_LL-F1-female_ = 9-10 mice.

Data was analyzed using two-tailed unpaired t test. *p < 0.05.


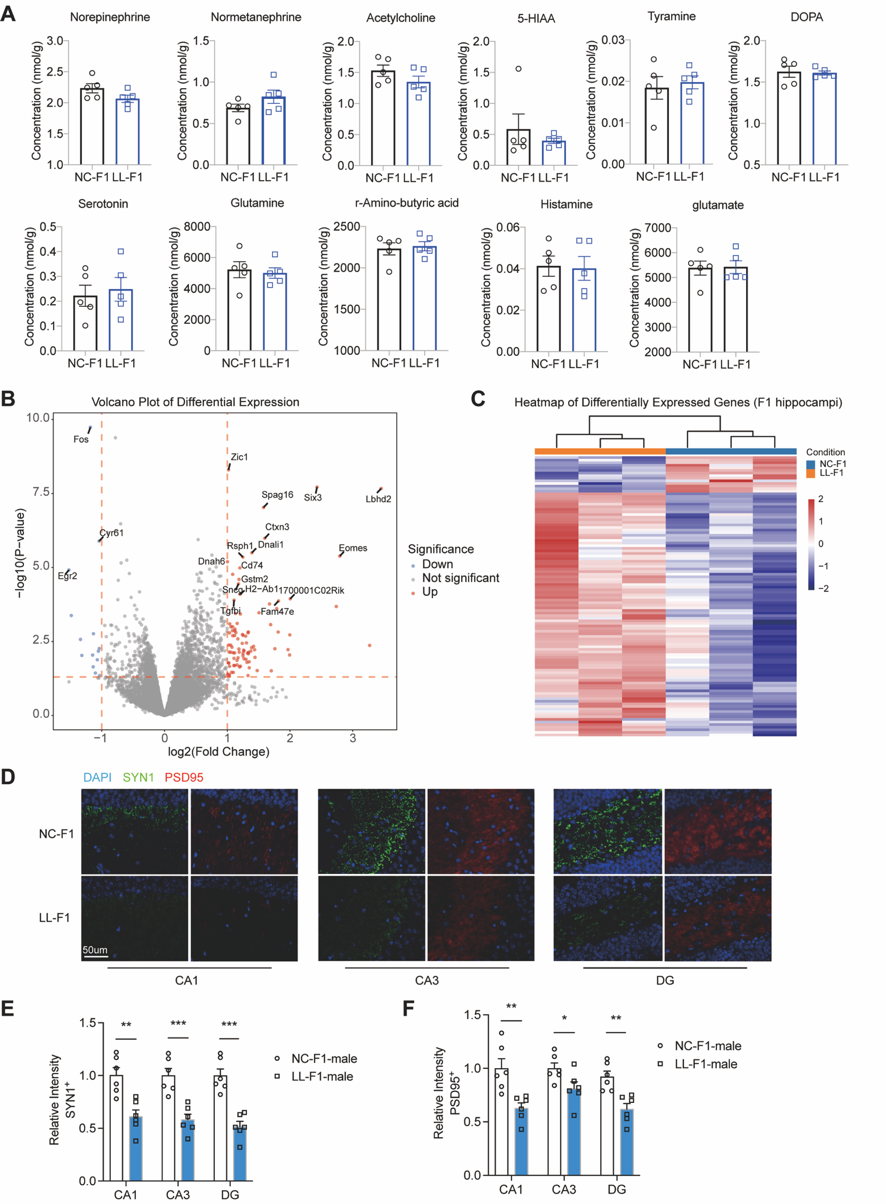


**Figure S2. Molecular and synaptic alterations in the hippocampus of F1 offspring.**

(A) Levels of neurotransmitters in the hippocampus of male offspring (n = 5 per group).

(B) Volcano plot of differentially expressed genes from F1 hippocampal RNA-seq (n = 3 per group).

(C) Heatmap showing normalized expression patterns of F1 hippocampal transcriptomes.

(D) Immunofluorescence staining of the hippocampal CA1, CA3, and DG regions from NC-F1 and LL-F1 mice. Nuclei were labeled with DAPI (blue), presynaptic terminals with SYN1 (green), and postsynaptic density structures with PSD95 (red). Scale bar, 50 μm.

(E, F) Relative fluorescence intensity of SYN1 and PSD95 were quantified using ImageJ from 3 mice per group, with two anatomically matched sections analyzed per mouse. Group comparisons were performed using unpaired two-tailed t-tests. Data was analyzed using two-tailed unpaired t test. *p < 0.05, **p < 0.01, ***p < 0.001.

**Figure S3. Validation of small RNA sequencing datasets.**

(A) Principal component analysis (PCA) of small RNA-seq samples. The plot shows the first two principal components, with mouse and human samples distinguished by color as indicated in the legend. Each dot represents one biological replicate.

(B) Gene Ontology enrichment analysis of predicted target genes of the 18 abundant upregulated sperm miRNAs identified in LL-F0 versus NC-F0 mice.

(C) Relative expression levels of sperm miRNAs were assessed by RT–qPCR in mouse and human samples. For mice, n_NC-F0_ = 6, n_LL-F0_ = 6 mice. For human, n _human-NC_ = 25, n _human-CD_ = 25 participants. Data was analyzed using two-tailed unpaired t test. **p* < 0.05, ***p* < 0.01.


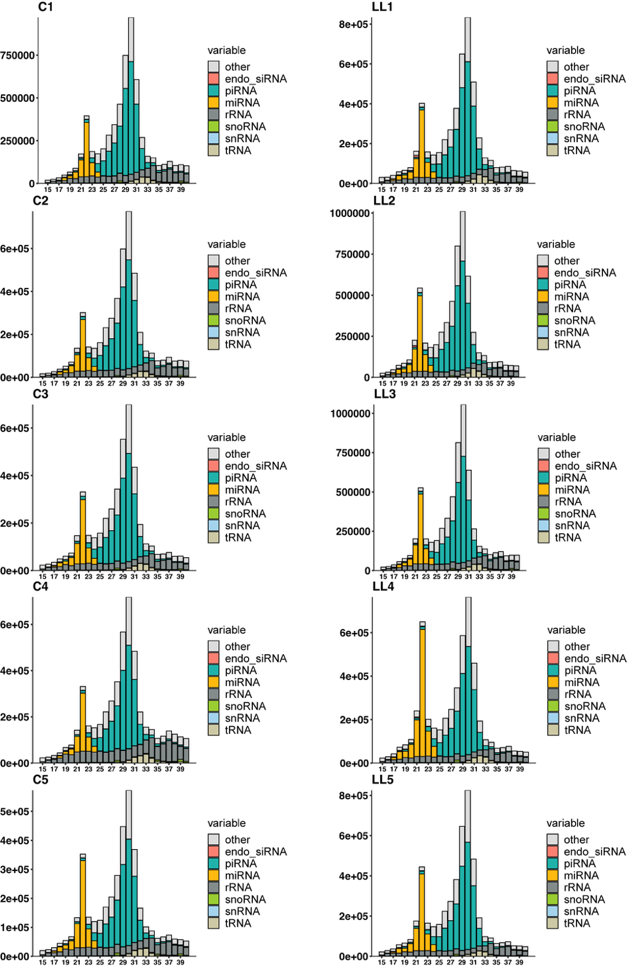


**Figure S4.** Length distribution of small RNAs in spermatogonial stem cells from NC-F0 and LL-F0 (n = 5 per group).


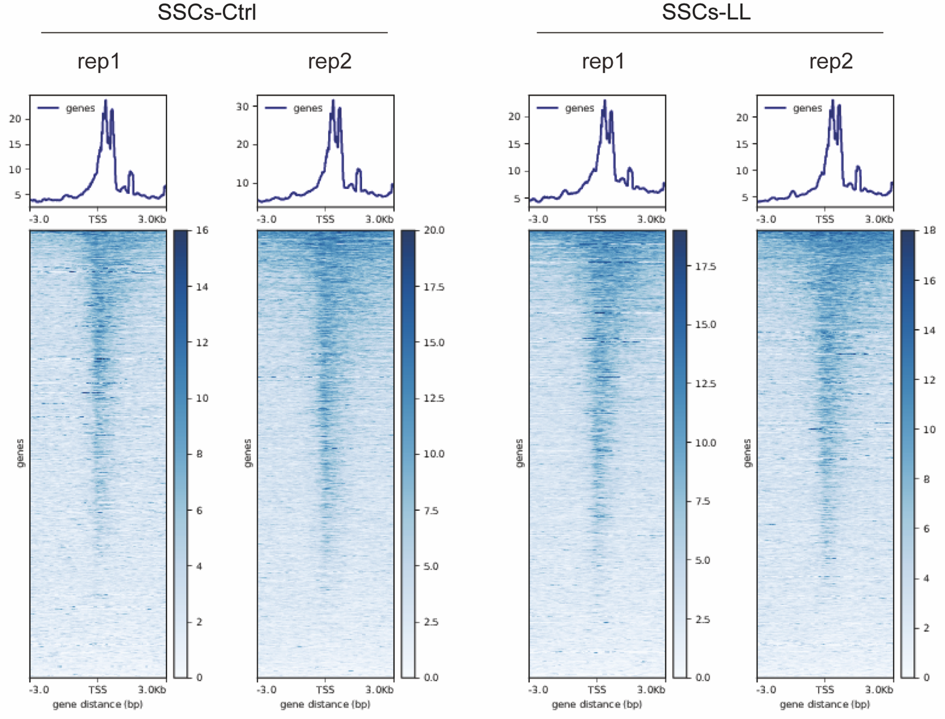


**Figure S5.** Transcription start site (TSS) enrichment heatmap of ATAC-seq signals in SSCs-Ctrl (n = 2) and SSCs-LL (n = 2).


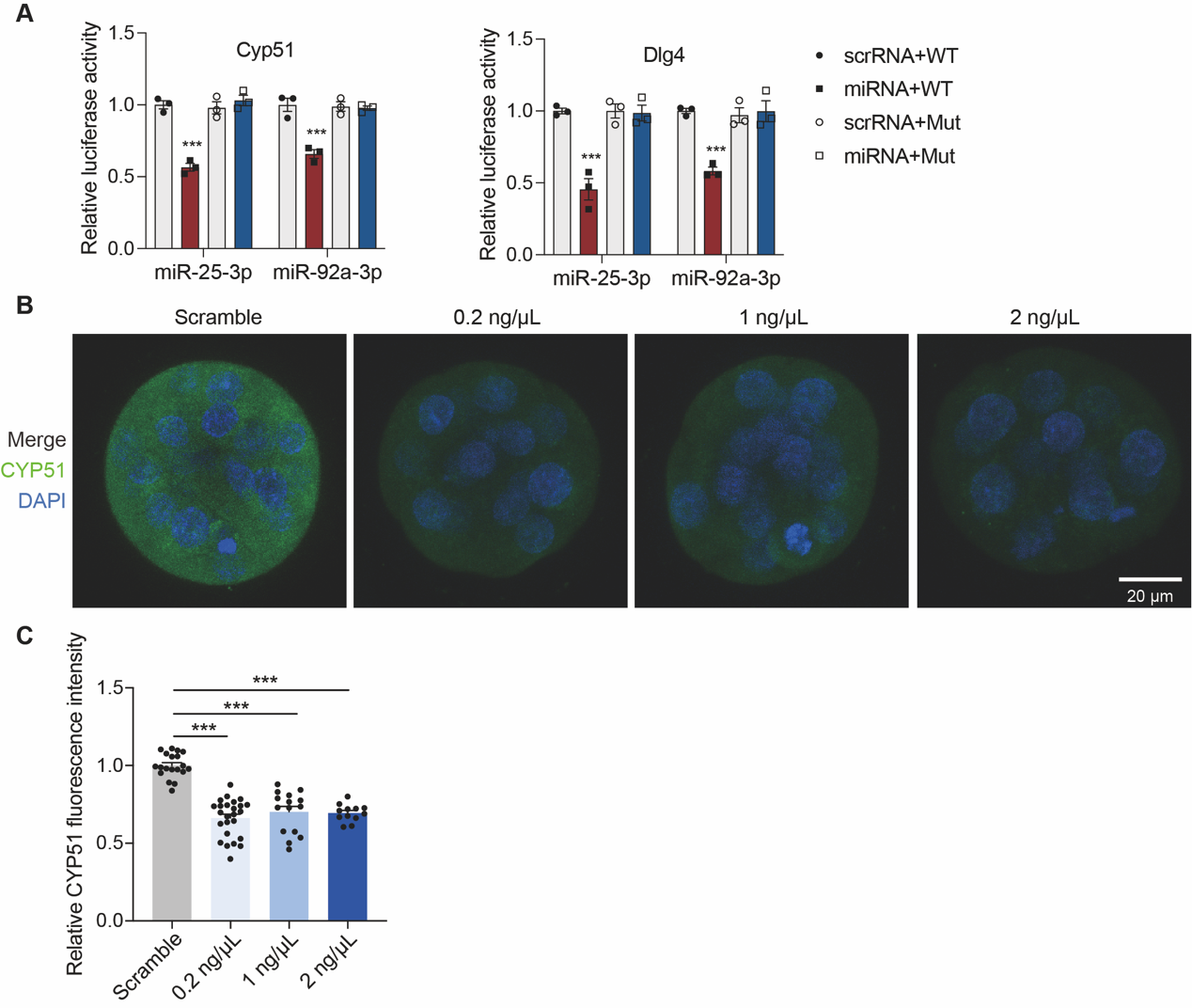


**Figure S6. miR-92a-3p and miR-25-3p directly target *Cyp51/Dlg4* and suppress embryonic CYP51 in a dose-dependent manner**

(A) Luciferase reporter assays validating that miR-92a-3p and miR-25-3p directly bind to the *Cyp51* and *Dlg4* 3′UTRs, respectively. WT and seed-mutant reporter constructs were co-transfected with miRNA expression plasmids in HEK293 cells (n = 3). Reduced luciferase activity in WT but not mutant reporters confirmed specific miRNA–3′UTR interactions.

(B, C) CYP51 immunofluorescence intensity was quantified in early morula-stage embryos injected with different concentrations of miRNA mimics. Embryos injected with scramble miRNA mimics served as the negative control. Fluorescence intensity was measured as mean pixel intensity within the embryo region after background subtraction and normalized to the scramble control group (Scramble = 1). Each dot represents one embryo (n = 12-25 per group). Data were obtained from three independent experiments.

Data are presented as mean ± SEM. Data were analyzed using one-way ANOVA followed by Tukey’s multiple comparisons test. ****p* < 0.001.

**Data S1**. Wheel-running activity, CLAMS metabolic profiling, RT–qPCR results, and corticosterone measurements in F0 males under circadian disruption.

**Data S2**. Raw behavioral datasets

**Data S3**. Differentially expressed genes (DEGs) from F1 hippocampal RNA-seq.

**Data S4**. Data for open field (OF) and elevated plus maze (EPM) following microinjection experiments.

**Data S5**. Quality control (QC) for small RNA sequencing of mouse and human sperm samples.

**Data S6**. DESeq2 output of mouse and human sperm small RNA seq.

**Data S7**. Target prediction and enrichment analysis of abundant upregulated sperm miRNAs

**Data S8**. miRNA target prediction results

**Data S9**. miRNA expression levels (RPM) in SSCs

**Data S10**. Uncropped and unprocessed Western blot images.

**Data S11**. Primer sequences used in this study.
