# Supplementary material for: Paternal Circadian Disruption Impairs Offspring Cognition via Sperm microRNAs
Source: Adv Sci (Weinh). 2026 Apr 28:e14510. Online ahead of print. doi: 10.1002/advs.202514510 (PMC13334623; doi:10.1002/advs.202514510)
Supplement: Supplementary file 2 — Supporting File 2: advs75462‐sup‐0002‐data.zip. [file ADVS-9999-e14510-s001.zip › advs75462-sup-0002-data/Data S1 F0 circadian disruption.pdf]

Supplement Data S1

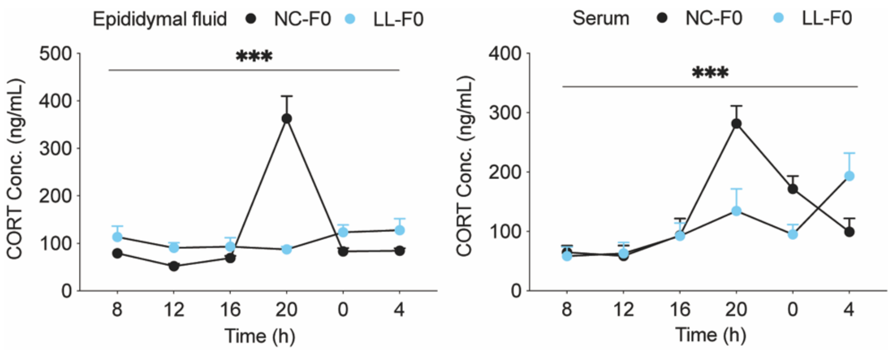

Data S1.1

Corticosterone concentrations were measured every 4 hours across a 24-h cycle at 08:00, 12:00, 16:00, 20:00, 00:00, and 04:00. $n_{NC\text{-}F0} = 5$, $n_{LL\text{-}F0} = 5$ mice each time point. Time is shown as clock time (h); 8 denotes 08:00. All data are presented as mean ± SEM. ***P < 0.001 by two-way repeated measures ANOVA with time × group interaction.

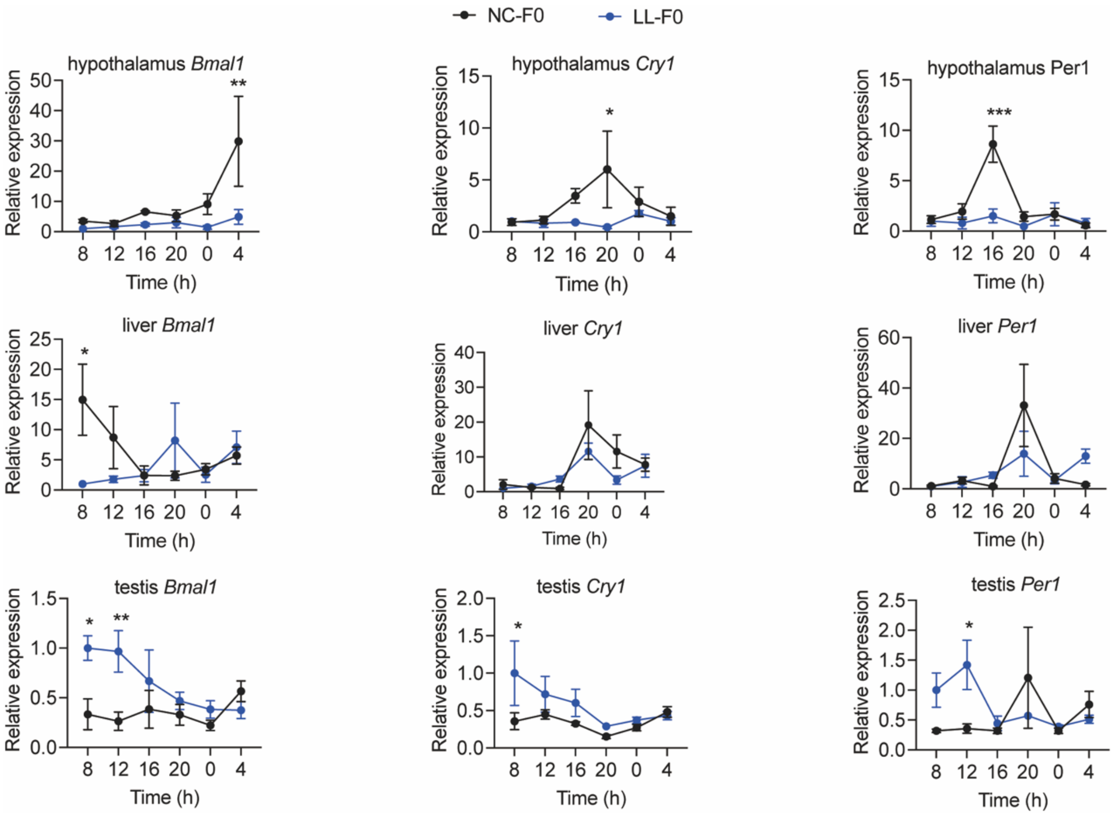

Data S1.2

qPCR analysis of *Bmal1*, *Cry1*, and *Per1* in hypothalamus, liver and testis. Relative expression was measured at 8, 12, 16, 20, 0, and 4 h in NC-F0 and LL-F0 mice (n = 4–5/time point). mRNA levels are expressed relative to the LL-F0 at 08:00 time point, defined as 1. Data are mean ± SEM. *p < 0.05, **p < 0.01, ***p < 0.001. Statistical analysis was performed using two-way ANOVA (Group × Time) followed by Bonferroni's multiple comparisons test.

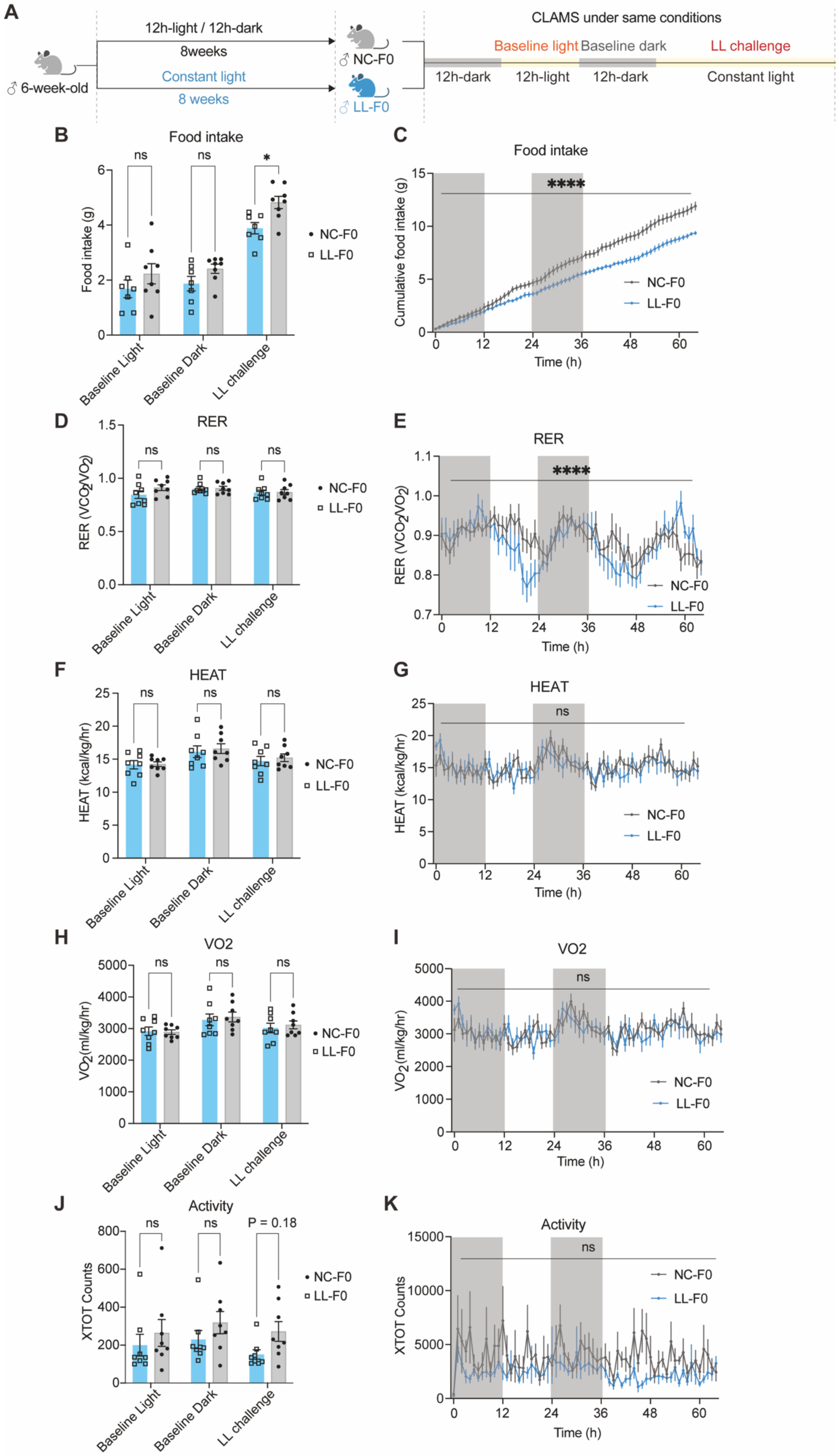
A
♂ 6-week-old
12h-light / 12h-dark
8weeks
Constant light
8 weeks
♂ NC-F0
♂ LL-F0
CLAMS under same conditions
Baseline light
Baseline dark
LL challenge
12h-dark
12h-light
12h-dark
Constant light
B
Food intake
Food intake (g)
ns
ns
*
NC-F0
LL-F0
Baseline Light
Baseline Dark
LL challenge
C
Food intake
Cumulative food intake (g)
****
Time (h)
D
RER
RER (VCO2/VO2)
E
F
HEAT
HEAT (kcal/kg/hr)
G
H
VO2
VO2(ml/kg/hr)
I
J
Activity
XTOT Counts
P = 0.18
K

Data S1.3
Mice were placed in a CLAMS apparatus under same lightning conditions (A) and monitored for food intake (B, C), RER (D, E), energy expenditure (F, G), oxygen consumption (H, I) and activity (J, K) over a 60-hr period. $n_{NC\text{-}F0} = 8$, $n_{LL\text{-}F0} = 7\text{-}8$ mice. Data were analyzed by two-way repeated measures ANOVA with time × group interaction (C, E, G, I, K), two-way ANOVA followed by Bonferroni's multiple comparisons test (B, D, F, H, J). **p* <0.05, *****p* < 0.0001.

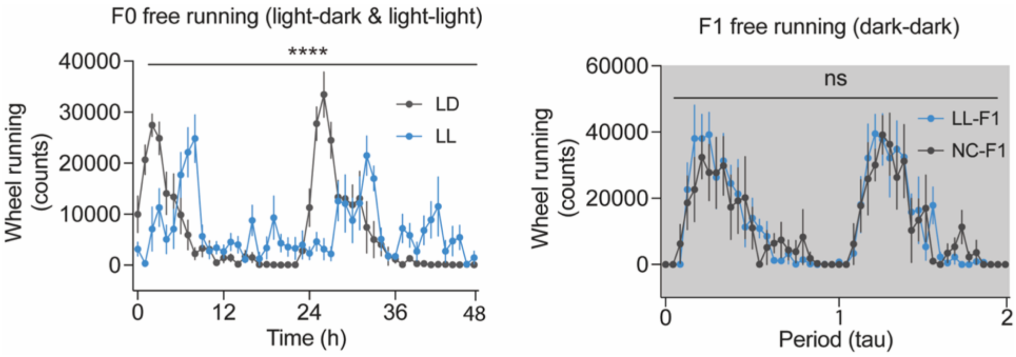

Data S1.4
Single animal locomotor activity of F0 and F1. $n_{LD} = 8$, $n_{LL} = 8$, $n_{NC\text{-}F1} = 5$, $n_{LL\text{-}F1} = 5$ mice. Data were analyzed by two-way repeated measures ANOVA with time × group interaction. ****p < 0.0001.
