# Supplementary material for: Paternal Circadian Disruption Impairs Offspring Cognition via Sperm microRNAs
Source: Adv Sci (Weinh). 2026 Apr 28:e14510. Online ahead of print. doi: 10.1002/advs.202514510 (PMC13334623; doi:10.1002/advs.202514510)
Supplement: Supplementary file 2 — Supporting File 2: advs75462‐sup‐0002‐data.zip. [file ADVS-9999-e14510-s001.zip › advs75462-sup-0002-data/Data S4 microinjection_OFT_EPM.pdf]

Supplement Data S4

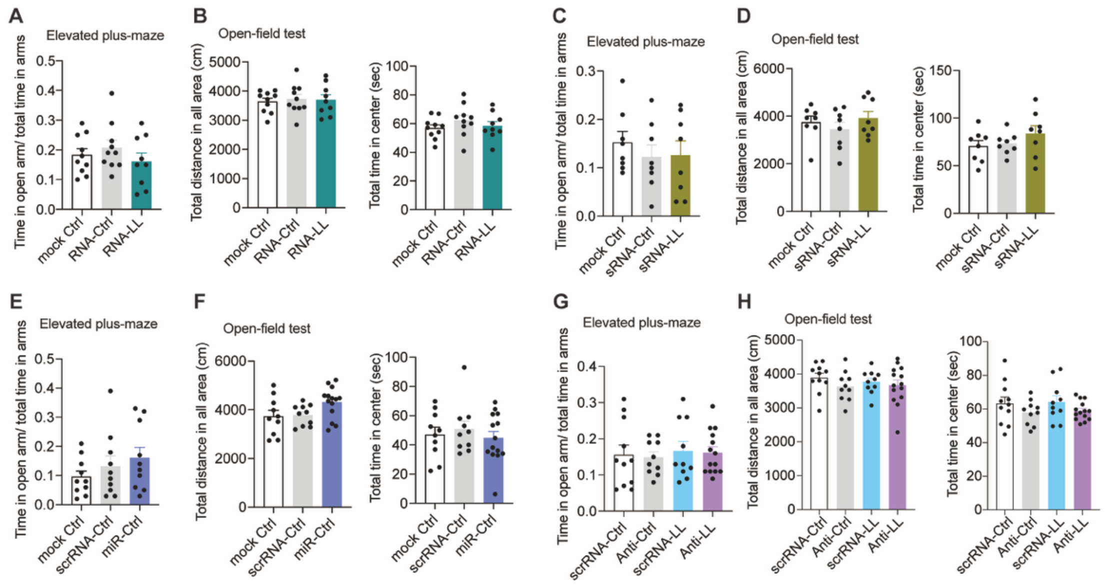

Data S4. (A, C, E, G) Elevated Plus Maze (EPM). Quantification of time spent in the open arms expressed as a proportion of total time spent in all arms (open arm time / total arm time). (B, D, F, H) Open Field Test (OFT). Quantification of total distance traveled and distance traveled within the center zone. $n_{\text{mock Ctrl}} = 10$, $n_{\text{RNA-Ctrl}} = 10$, $n_{\text{RNA-LL}} = 9$ mice. $n_{\text{mock Ctrl}} = 8$, $n_{\text{sRNA-Ctrl}} = 8$, $n_{\text{sRNA-LL}} = 8$ mice. $n_{\text{mock Ctrl}} = 10$, $n_{\text{scrRNA-Ctrl}} = 10$, $n_{\text{miR-Ctrl}} = 10\text{-}14$ mice. $n_{\text{scrRNA-Ctrl}} = 11$, $n_{\text{anti-Ctrl}} = 10$, $n_{\text{scrRNA-LL}} = 10$ mice, $n_{\text{anti-LL}} = 14$ mice. Data are presented as mean ± SEM.
