# Supplementary material for: Paternal Circadian Disruption Impairs Offspring Cognition via Sperm microRNAs
Source: Adv Sci (Weinh). 2026 Apr 28:e14510. Online ahead of print. doi: 10.1002/advs.202514510 (PMC13334623; doi:10.1002/advs.202514510)

Supplement Data S5

mouse sperm small RNAseq QC (NC-F0 n=7)

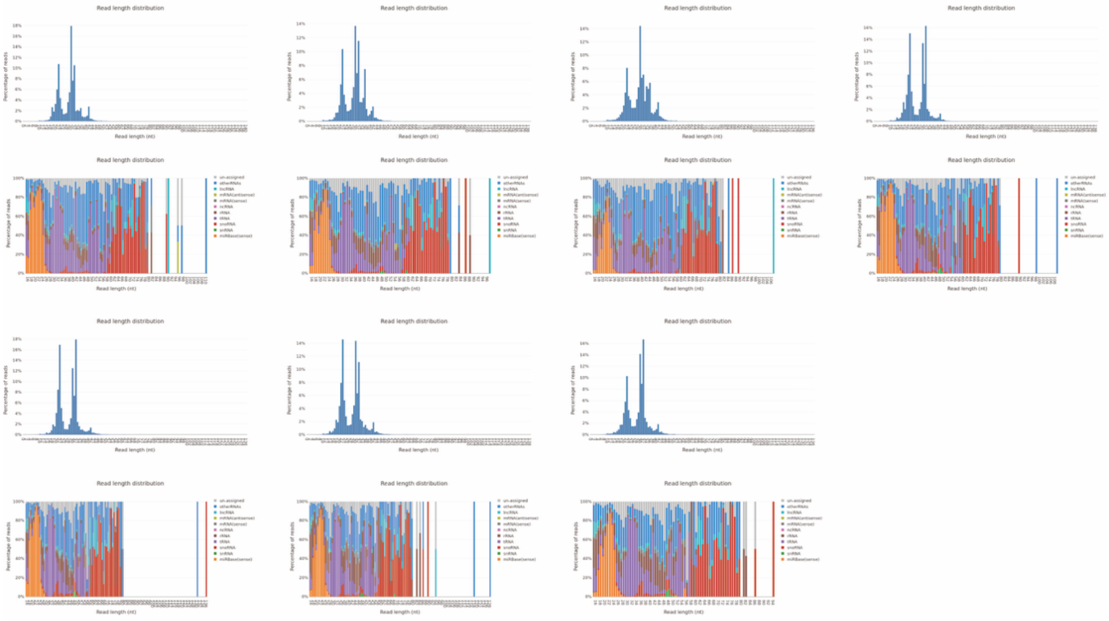

mouse sperm small RNAseq QC (LL-F0 n=7)

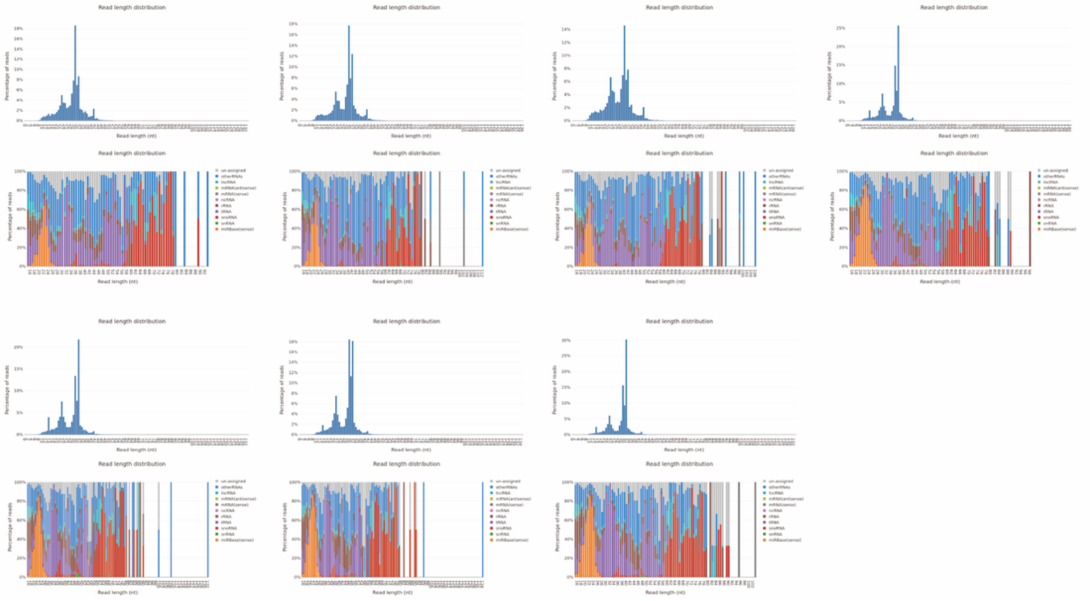

human sperm small RNAseq QC (human-NC n=10)

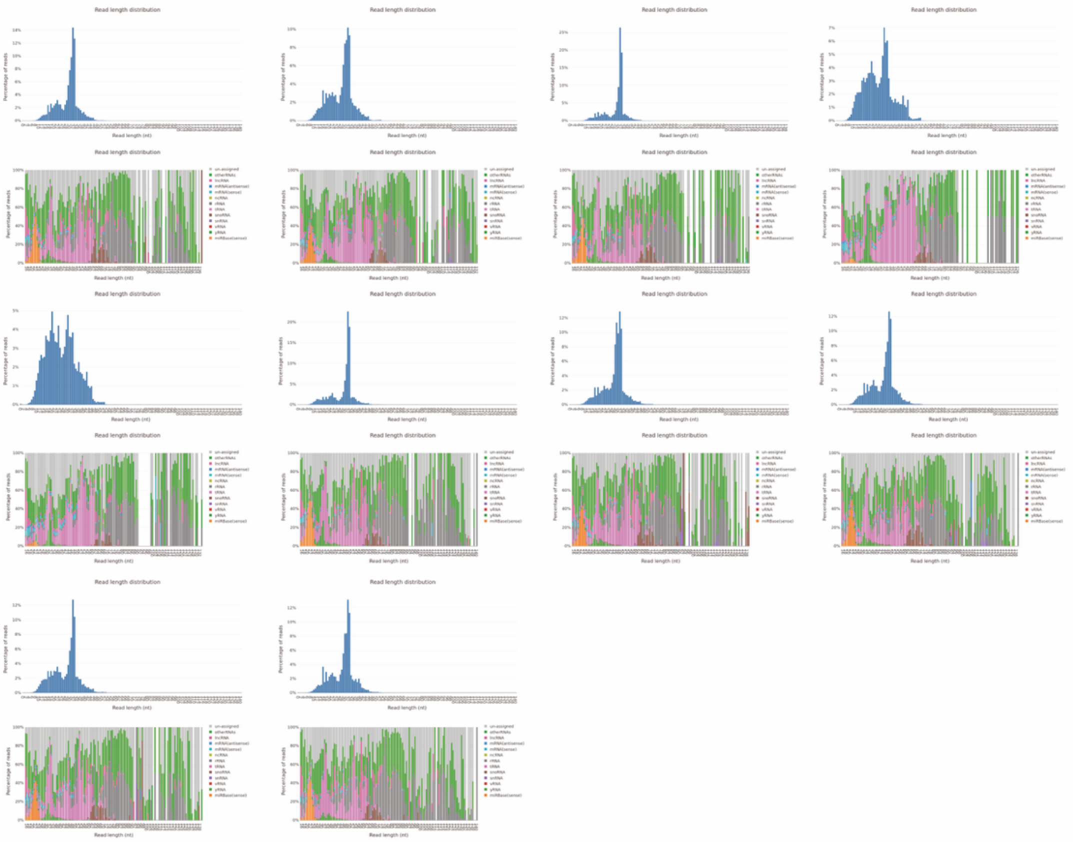

human sperm small RNAseq QC (human-CD n=10)

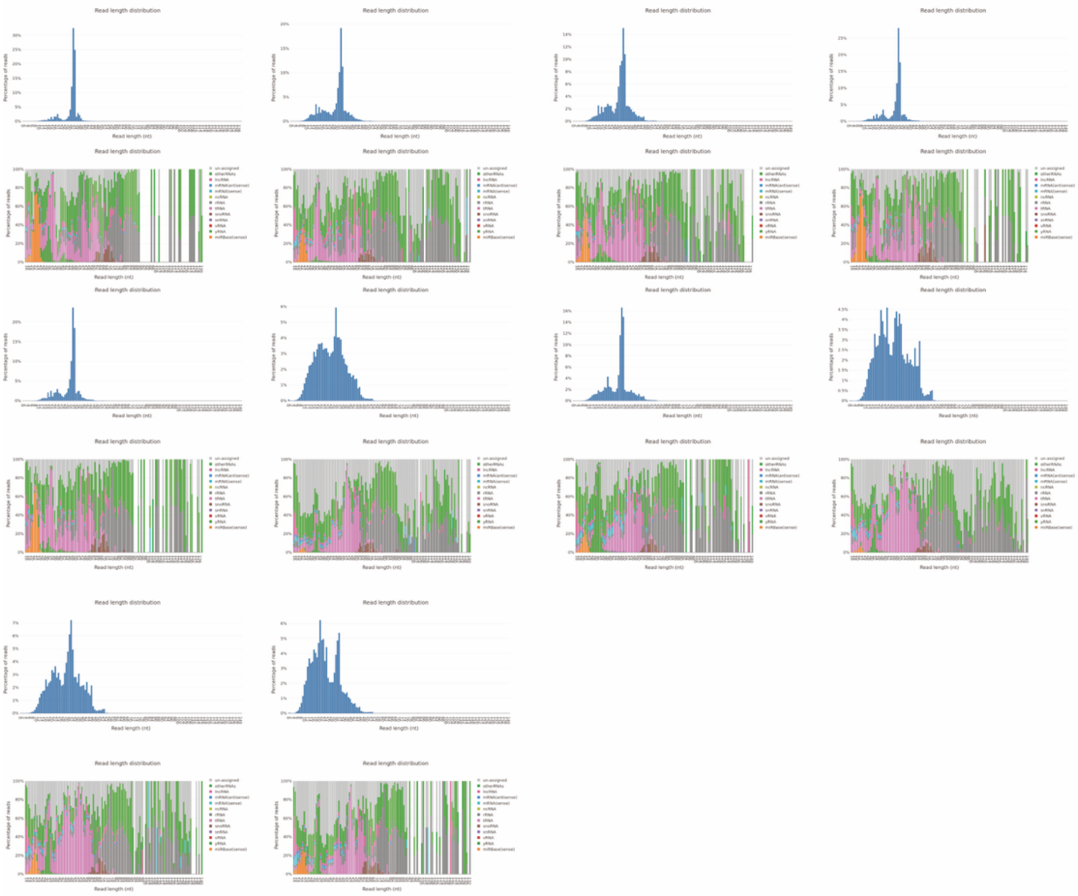

Supplement: Supplementary file 2 — Supporting File 2: advs75462‐sup‐0002‐data.zip. [file ADVS-9999-e14510-s001.zip › advs75462-sup-0002-data/Data S5 QC_smallRNA_length_distribution.pdf]
