# Supplementary figures and images for: Paternal Circadian Disruption Impairs Offspring Cognition via Sperm microRNAs
Source: Adv Sci (Weinh). 2026 Apr 28:e14510. Online ahead of print. doi: 10.1002/advs.202514510 (PMC13334623; doi:10.1002/advs.202514510)

Source\_DATA\_Extended Data Fig. 2

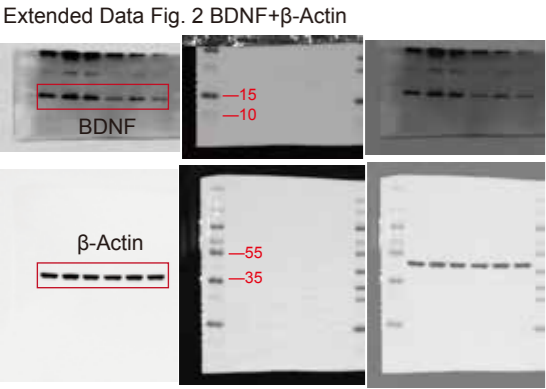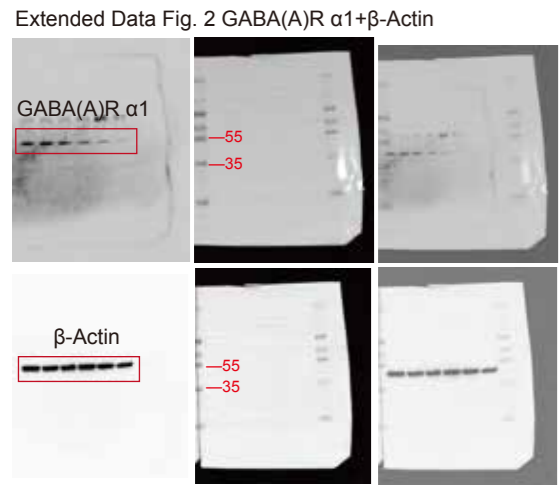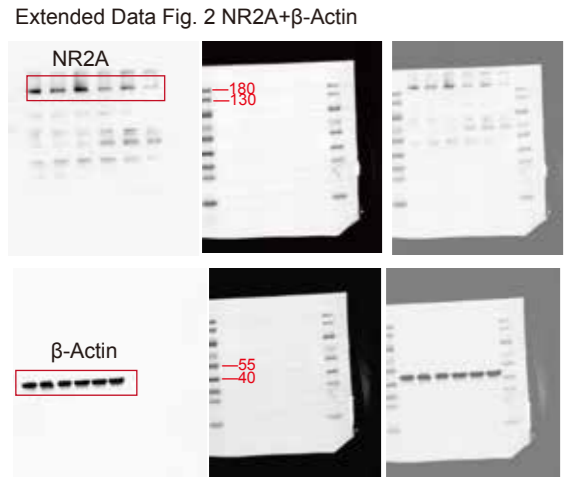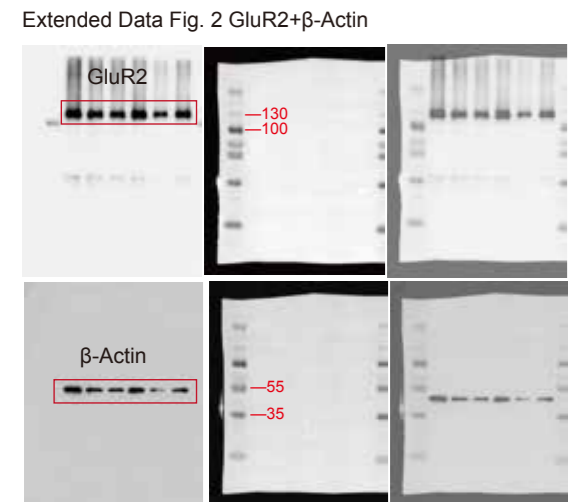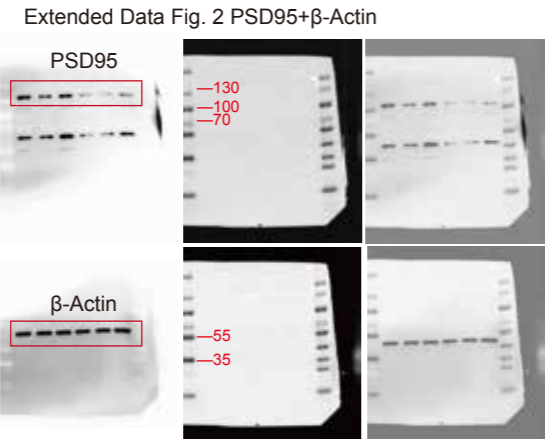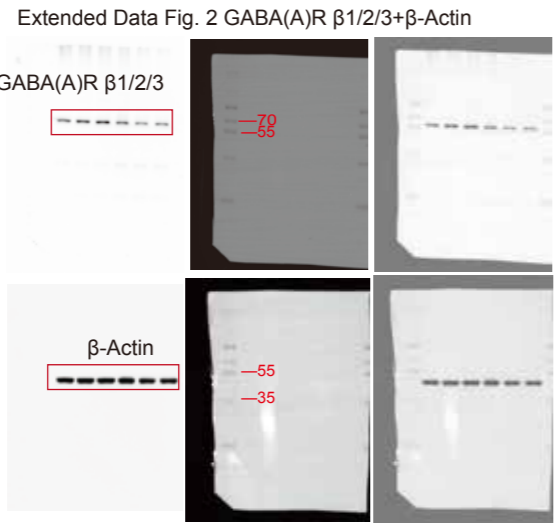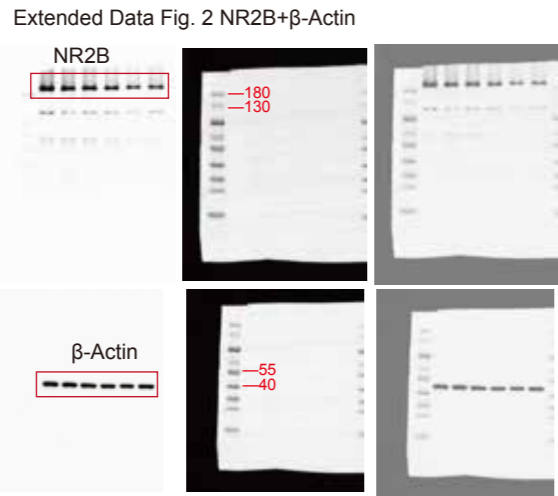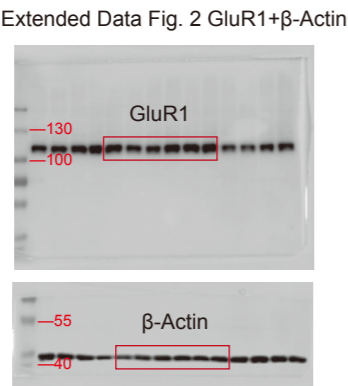

Source\_DATA\_Extended Data Fig. 3D

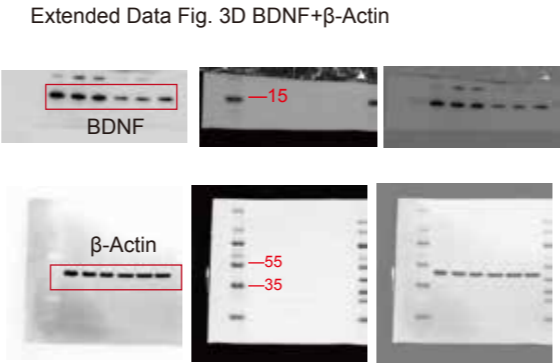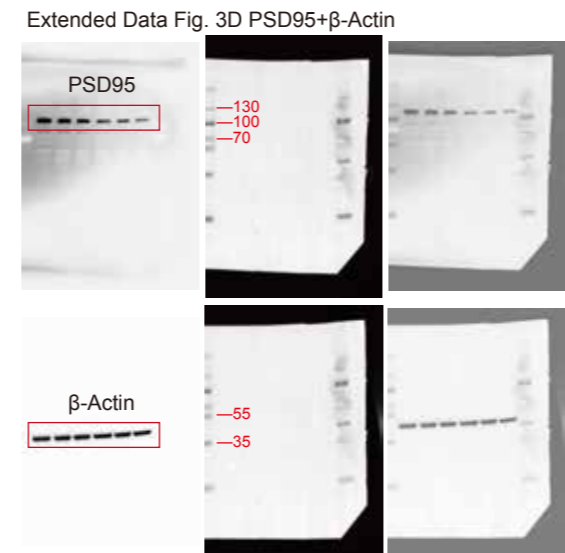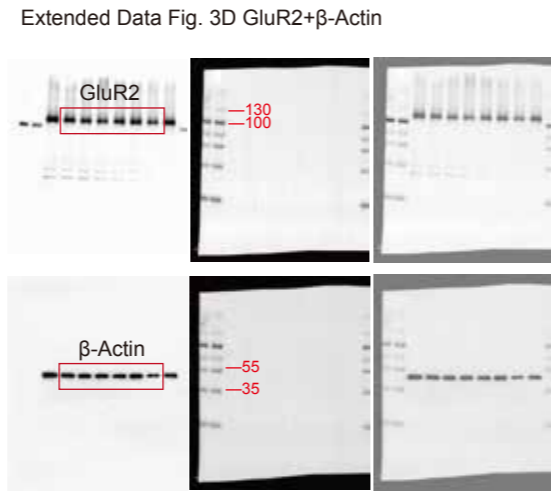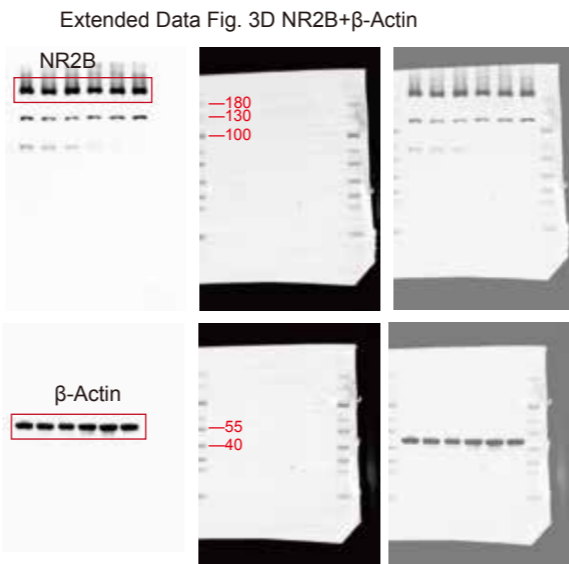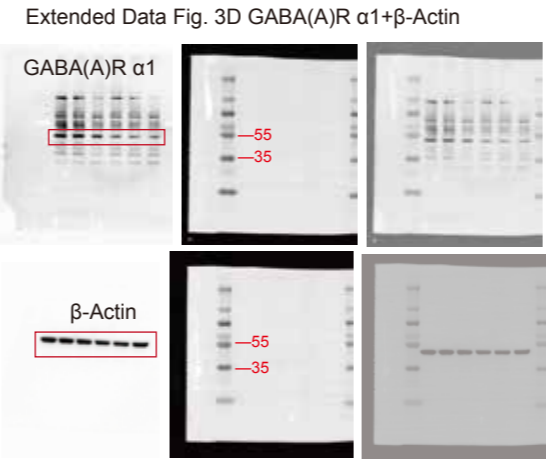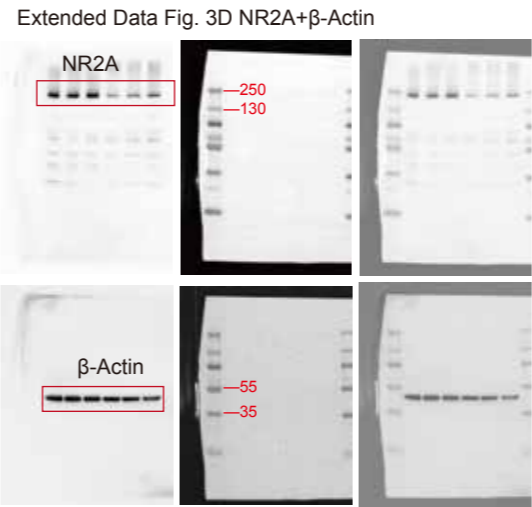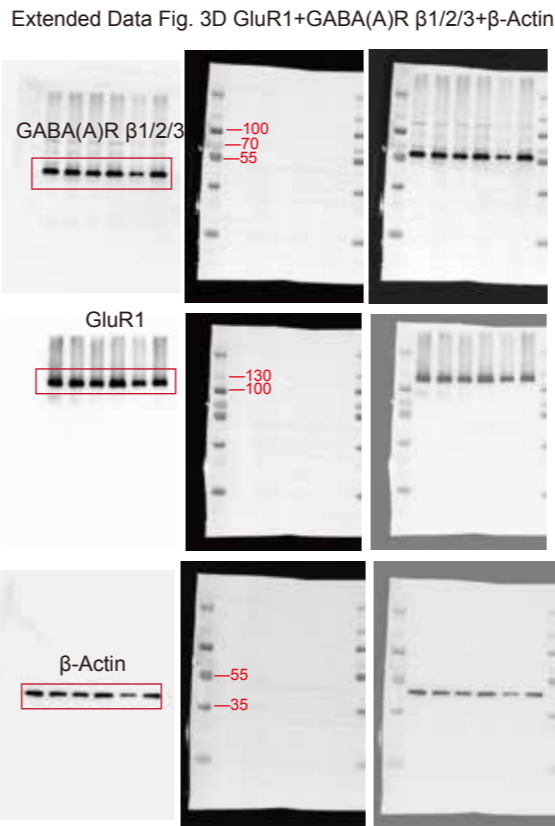

Source\_DATA\_Extended Data Fig. 3H

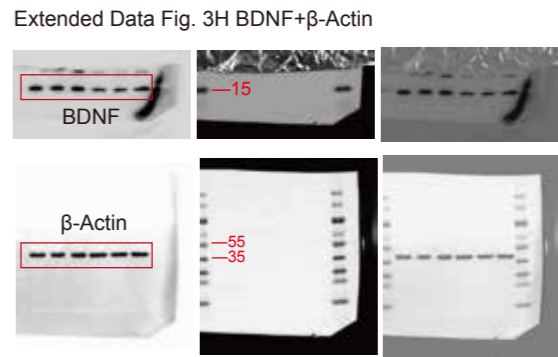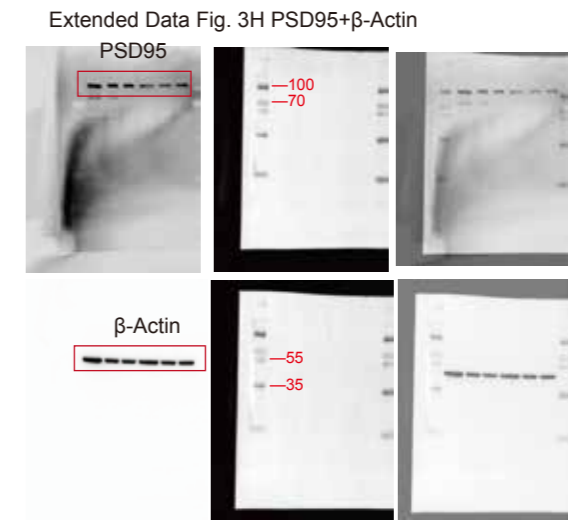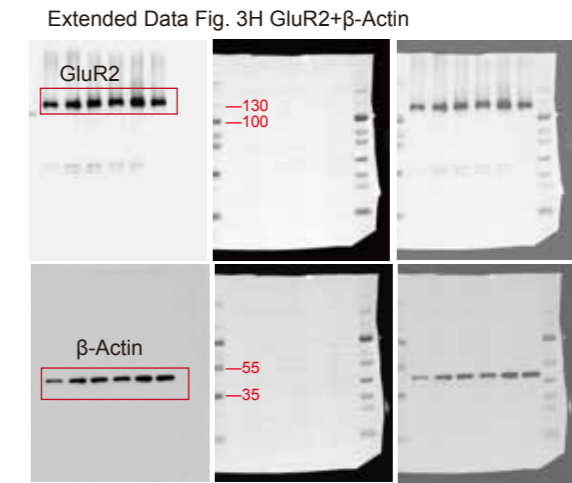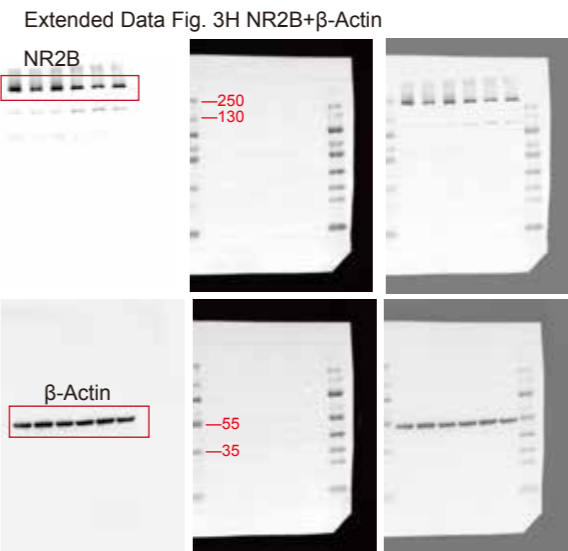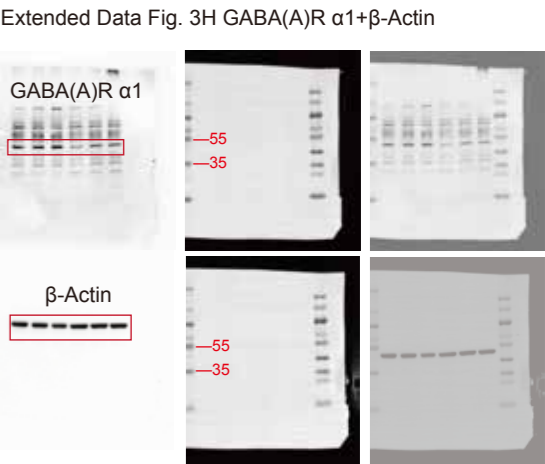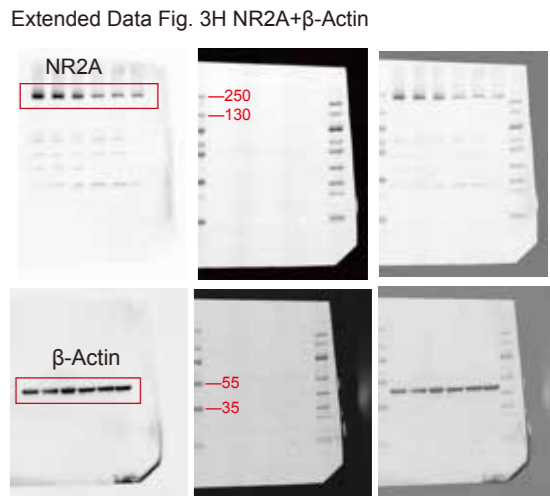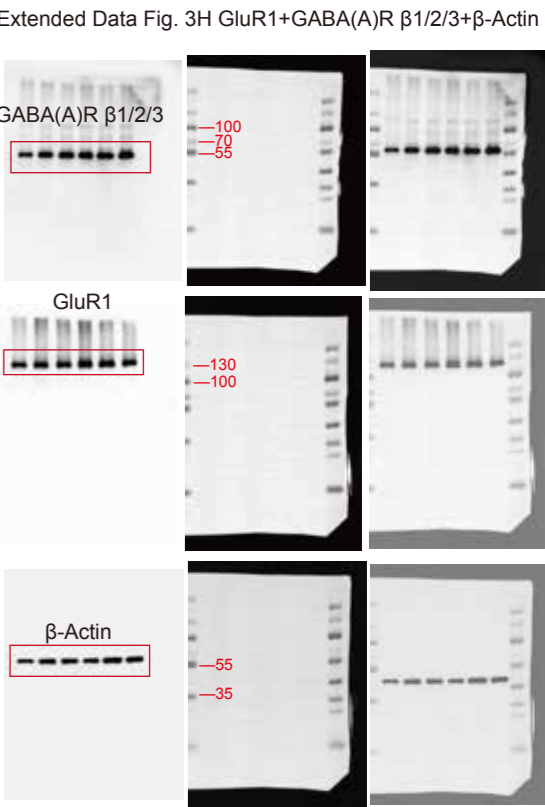

Supplement: Supplementary file 2 — Supporting File 2: advs75462‐sup‐0002‐data.zip. [file ADVS-9999-e14510-s001.zip › advs75462-sup-0002-data/Data S10 WB source data.pdf]
